# A systematic literature review on integrating AI-powered smart glasses into digital health management for proactive healthcare solutions

**DOI:** 10.1038/s41746-025-01715-x

**Published:** 2025-07-05

**Authors:** Boyuan Wang, Ying Zheng, Xihao Han, Liang Kong, Gexin Xiao, Zunxiong Xiao, Shanji Chen

**Affiliations:** 1 Beijing Xiaotangshan Hospital, Beijing, China; 2 Department of Biomedical Sciences, City University of Hong Kong, Hong Kong, China; 3 School of Mathematical Sciences, Capital Normal University, Beijing, China; 4 National Institute of Hospital Administration (NIHA), Beijing, China; 5 The First Affiliated Hospital of Hunan University of Medicine, Huaihua, China; 6 Hunan University of Medicine, Huaihua, China; 7 Hunan Primary Digital Engineering Technology Research Center for Medical Prevention and Treatment, Huaihua, China

**Keywords:** Information technology, Lifestyle modification, Preventive medicine

## Abstract

AI-powered smart glasses are emerging as a highly promising advancement in the field of digital health management, owing to their capabilities in real-time monitoring, chronic disease management, and personalized treatment planning. To comprehensively understand the current state of development, we systematically searched multiple databases, including Web of Science, PubMed, and IEEE Xplore, to collect relevant literature. This paper provides a systematic analysis of the current applications of smart glasses in healthcare, focusing on their potential benefits and limitations. Key issues discussed include user engagement, treatment adherence, data privacy, standardization, battery efficiency, clinical validation, and medical ethics. Our findings suggest that, supported by emerging clinical evidence, smart glasses have demonstrated significant improvements in areas such as assisted medical services, health management, anxiety alleviation in children, and telemedicine. By integrating multi-modal sensors, these devices are capable of accurately tracking certain physiological indicators and synchronizing real-time visual input, thereby enhancing the accuracy and timeliness of health interventions and medical services. Notably, some cutting-edge smart glasses have adopted advanced artificial intelligence algorithms, particularly large language models (LLMs) with context awareness and human-like interaction capabilities. These AI-powered glasses can offer real-time, personalized dietary and health management recommendations tailored to users’ daily life scenarios. Building on these findings, this study further proposes a conceptual framework for proactive health management using smart glasses and explores future directions in technological development and practical applications. Overall, AI-enhanced smart glasses show great potential as a critical interface between healthcare providers and patients, poised to play a vital role in the future of personalized medicine and continuous health management.

## Introduction

Wearable devices, broadly defined as technologies designed to be worn on or attached to the human body, embody a practical realization of advanced wearable technology. These devices function as seamless extensions of personal space, operating under the user’s control to facilitate continuous interaction and functionality^1,2^. In recent years, with the advancement of artificial intelligence (AI) technologies, wearable devices have become an essential tool in health management. Google’s Verily Life Sciences has launched the four-year Project Baseline^3^, which aims to recruit 10,000 participants to integrate data from wearable devices and genetic testing, with the goal of predicting emergencies such as strokes and seizures. Similarly, the All of Us research initiative by the National Institutes of Health (NIH) also utilizes wearable devices to collect physiological data, advancing precision medicine^4^. Both studies leverage real-time health data collection via wearables to enable personalized interventions and early detection. Among the array of mainstream wearable devices—including smartwatches, fitness trackers, wearable cameras, and medical wearables—smart glasses have emerged as a particularly promising innovation, especially within the domain of health management^5,6^.Unlike other types of wearables, AI smart glasses integrate advanced sensors and algorithms to monitor physiological data in real-time, providing instant feedback and health recommendations^7^. Meanwhile, the high speed and low-latency features provided by 5G networks, combined with the capabilities of edge computing, can achieve real-time health monitoring and immediate feedback, greatly enhancing the efficiency and security of telemedicine^8^.

The integration of miniaturization, sensor technology, and artificial intelligence (AI) has revolutionized the landscape of wearable devices, positioning AI-powered smart glasses as a cornerstone innovation in health management. These advanced wearables combine compact design with portability, addressing the escalating global demand for continuous, real-world health monitoring and personalized care. The ongoing development of this technology is expected to significantly advance real-time health surveillance, preventive medicine, precision healthcare, and tailored interventions^9^.

Globally, the challenges in managing chronic diseases are pronounced^10^, characterized by fragmented service delivery networks, insufficient primary care infrastructure, inefficient funding mechanisms, and underdeveloped health information systems. These systemic issues impede the effectiveness of health management programs and contribute to inequities in access to timely and quality care. In resource-constrained settings, these barriers are even more pronounced, delaying early intervention and exacerbating health disparities^11–13^.

AI-powered smart glasses offer a transformative solution to these challenges^14^. Integrated AI algorithms can process real-time physiological data to provide users with personalized health recommendations, early warnings of potential issues, and actionable insights that emphasize prevention and prediction in health management^15^. The lightweight and unobtrusive nature of smart glasses ensures they can be seamlessly integrated into daily life, allowing for constant health tracking without disrupting routine activities^16^. Moreover, their potential in advancing remote diagnostics and treatment is significant. Smart glasses can facilitate telemedicine, enabling healthcare providers to remotely assess patients and deliver timely interventions, which is especially critical in addressing healthcare disparities in underserved regions worldwide^17^.

Therefore, this paper focuses on the application of AI-powered smart glasses in health management, systematically reviewing their key technological pathways, clinical application scenarios, and future development trends. It emphasizes their strategic value in advancing global health equity and enabling precision health management.

**Fig. 1:**
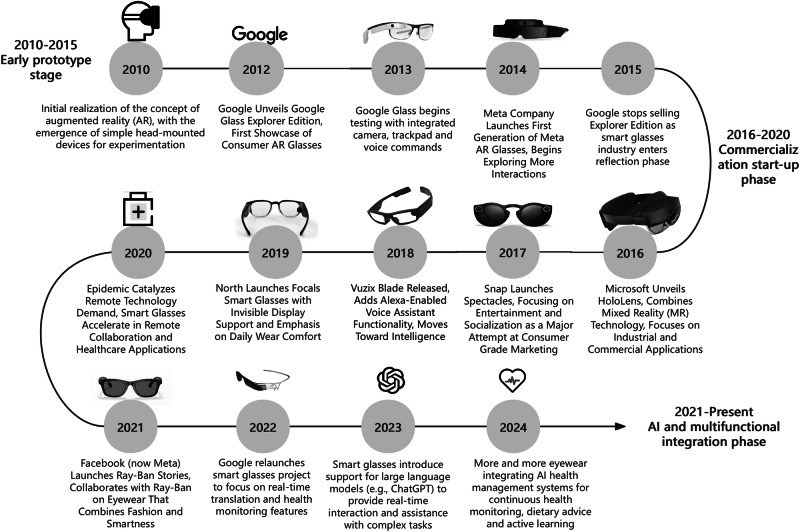
Historical development of smart glasses. Smart glasses are developing rapidly year by year, and the development forms are becoming more diversified. This timeline highlights selected representative smart glasses products based on technological innovation and market influence. It does not include all existing products.

AI-powered smart glasses, as an advanced evolution of wearable technology, increasingly incorporate a range of cutting-edge technologies, including Augmented Reality (AR), Virtual Reality (VR), and Mixed Reality (MR), to deliver immersive and interactive user experiences. The distinctive characteristics and synergistic advantages of these technologies have led to their widespread adoption across multiple application scenarios such as entertainment, gaming, education, and professional training domains. For instance, the combination of educational theory and virtual reality (VR) technology is feasible. By using VR technology, students’ participation and learning outcomes can be significantly enhanced, especially in experimental or practical courses, to improve the educational experience^18^. The combination of VR technology and educational theory has great development potential. It changes the traditional way of learning and provides a new direction for future medical educational practice^19^. In addition, their exceptional capability for real-time health monitoring establishes them as a transformative tool in modern healthcare applications^20^. To better understand the historical development and current technological status of smart glasses, Fig. 1 provides an evolution from early prototypes to modern products. It is noteworthy that while there are various types of smart glasses available on the market, our research specifically focuses on advanced models integrated with large language models (LLMs), augmented reality (AR), and other key functionalities, as they represent the forefront of technological advancements and show immense potential in healthcare. For instance, MYVU AR glasses and Rokid glasses not only support LLMs but also feature image object detection capabilities, enabling them to perform highly specialized tasks in complex medical environments.

During the Prototype Phase, the inception of smart glasses can be traced back to the pioneering efforts of Professor Steve Mann, widely acknowledged as the “Father of Wearable Computing.” Mann’s groundbreaking work during this era resulted in the development of early prototypes that primarily integrated Augmented Reality (AR) functionalities, laying the foundational stones for subsequent advancements in smart eyewear technology. However, owing to their considerable size and the experimental phase of the technology, these early devices were largely restricted to academic research environments. One of Mann’s contributions was the EyeTap apparatus, which achieved a milestone by capturing the wearer’s visual perspective while processing and overlaying digital information. Although the EyeTap represented a notable leap forward in technology, its practical applications were limited by the bulky design and the experimental state of the technology, thereby confining its usage mainly to academic research within laboratory settings, rather than achieving widespread adoption in daily life.

In the Commercialization Initiation Phase (2010–2015), advancements in hardware and technology facilitated the emergence of simpler head-up displays (HUDs) in the early 2010s^21^, which gradually found applications in military, industrial, and scientific research fields. In 2012, Google launched the Google Glass Explorer Edition, marking the beginning of modern smart glasses with AR features. By 2013, Google Glass^22^ incorporated a camera, touch-pad, and voice commands, offering users an entirely new way to access information. However, due to concerns over privacy, social acceptance, and battery life, Google Glass did not achieve commercial success but provided valuable lessons for future innovations. Meanwhile, Japan’s NTT Docomo demonstrated AR Walker in 2010^23^, focusing on AR functionalities which laid the groundwork for subsequent developments. Although smart glasses had not yet been widely applied to health monitoring or healthcare, there were preliminary attempts. For instance, Google Glass was used to assist doctors in surgical guidance and teleconsultation^22^. Additionally, it helped children with autism recognize facial expressions^24^, indicating its potential in education^25^ and healthcare^14^.

The Diversification and AI Integration Phase (2016–2020) witnessed the maturation of smart glasses, introducing more diverse application scenarios and technological breakthroughs. Commencing in 2016, this era saw Snap Inc., the parent company of Snapchat, launch the Spectacles series, captivating a younger audience with its distinctive video recording capabilities. Additionally, the release of the Vuzix Blade in 2018, which integrated the Alexa voice assistant, further propelled consumer adoption by enhancing user interaction and functionality. The collaboration between Ray-Ban and Meta produced the Stories smart glasses, popular for photography, videography, and audio playback, designed to seamlessly integrate into daily life. Microsoft’s HoloLens^26^, launched in 2016, combined mixed reality (MR) technology, targeting industrial and professional applications. In other parts of Asia, Samsung Electronics actively participated in the competition, launching Galaxy Glass to connect smartphones with personal wearables. Sony focused on developing specialized smart glasses for industries like medical training and remote collaboration. During this phase, smart glasses saw significant growth in healthcare applications. Microsoft HoloLens has been utilized in surgical training, enhancing doctors’ learning efficiency and operational accuracy through three-dimensional views. Moreover, smart glasses entered public health sectors, such as supporting fever screening at Liangzhu Museum and Liangzhu Ancient City Ruins in Hangzhou, China, using non-contact temperature detection methods^27^. This approach reduced virus transmission risks while improving operational efficiency.

The New Era of Smart Glasses as AI Terminals (Since 2021) has been characterized by the integration of advanced AI and machine learning technologies, enabling the development of increasingly sophisticated models^28^. Rokid Glasses exemplify this trend with transparent lenses, stylish design, and built-in displays, providing real-time translation and navigation services via AI vision perception, greatly enhancing the user experience. XREAL Air AR glasses have gained popularity for their immersive virtual screen experiences^29^. Tech giants in the U.S., including Amazon and Meta Platforms (formerly Facebook), have increased investment in smart glasses, releasing some advanced and user-friendly products. Amazon’s Echo Frames^30^ allow users to access various information and services through simple voice commands. Meta’s Ray-Ban Stories emphasize social sharing, enabling users to easily capture memorable moments^31^. In China, Baidu is slated to launch its Xiaodu AI Glasses in the first half of 2025, marking a significant milestone as they are touted as “the world’s first AI glasses equipped with a native Chinese model^32^.” Designed by Baidu’s hardware division, Xiaodu Technology, these glasses showcase Baidu’s advanced Ernie language model, aiming to integrate AI into wearable technology tailored for Chinese-speaking users. Weighing a mere 45 grams, the Xiaodu AI Glasses feature a 16MP ultra-wide-angle camera and AI image stabilization technology, supporting smooth and stable first-person perspective photography. Additionally, the glasses are equipped with a four-microphone array for effective sound capture and open-back leak-proof speakers for clear audio, providing users with an immersive, hands-free experience. The glasses support six core functions: real-time Q&A, calorie recognition, object recognition through an encyclopedia feature, audio-visual translation, and smart reminders. Notably, since 2021, smart glasses have increasingly integrated AI technology to offer personalized user experiences. In 2022, Google revived its smart glasses project, focusing on real-time translation and health monitoring. That same year, Large Language Models like ChatGPT were introduced into smart glasses, providing real-time interaction and support for complex tasks^33,34^.

The evolution of smart glasses reflects the close cooperation and intense competition among different countries and regions under globalization. Each country leverages its own technological strengths and development needs to jointly advance this emerging technology, forming a vibrant international market landscape. As technology continues to evolve and societal acceptance of smart wearable devices grows, smart glasses are expected to become an indispensable part of everyday life, transforming how we interact with the world.

The development of AI-powered smart glasses is characterized by rapid technological advancements and a growing diversification of applications. These devices integrate cutting-edge sensor technologies with AI algorithms, including deep learning and computer vision, to significantly enhance their data processing and analytical capabilities.

The rapid expansion of the global AI industry underscores the burgeoning growth of AI-powered smart glasses^35–37^. Notably, leading nations such as the United States and China^38^ are at the forefront in terms of enterprise density and investment scale. These nations are instrumental in fostering innovation and propelling the market adoption of AI-driven products, including smart glasses. A prime example is the Ray-Ban Meta smart glasses, a collaborative venture between Meta and Ray-Ban. These glasses have achieved significant market success, with over 1 million units sold by May 2024^39^. However, despite the swift pace of technological advancements, several challenges persist. Key issues include:

Data privacy concerns, the continuous collection and processing of sensitive health data, necessitate robust privacy safeguards. As smart glasses become more integrated into daily life, ensuring the secure handling of personal data is paramount.

User experience optimization, ensuring seamless interaction and comfort for diverse user demographics, remains an ongoing challenge. The design and functionality of smart glasses must accommodate a wide range of user needs and preferences to enhance adoption.

Lack of standardized frameworks, the absence of universally accepted technical standards impede interoperability and widespread adoption. Standardization is crucial for the development of a cohesive ecosystem that supports the seamless integration of smart glasses with other devices and systems.

Despite these challenges, the maturation of foundational technologies and the expansion of application scenarios are unlocking significant market potential for AI-powered smart glasses^40^. As industry evolves, these devices are poised to become a driving force of innovation, translating technological breakthroughs into practical solutions across healthcare, industrial, and consumer sectors.

This study makes several contributions to the field of digital health management. To the best of our knowledge, this study represents the first systematic review focusing on the application of AI-powered smart glasses in the field of health management. Following the PRISMA guidelines, we conducted a Systematic Literature Review (SLR)^41^ to comprehensively examine recent advancements in AI-driven smart glasses, with a particular focus on their integration into digital health management systems aimed at facilitating proactive healthcare interventions. Secondly, through an in-depth analysis of the existing literature, we explored the core strengths and challenges associated with smart glasses in healthcare contexts. Key issues addressed include user engagement, treatment adherence, data privacy protection, standardization, battery efficiency, clinical validation, and medical ethics. Based on these findings, we propose corresponding strategies for improvement and future research directions. Thirdly, we highlight the potential of AI-enabled smart glasses to enhance clinical workflows, increase patient engagement, and improve treatment compliance. These findings demonstrate the unique value of this technology in advancing personalized medicine and precision health management.

The remainder of this paper is organized as follows: The “Results” section summarizes the key findings of our review, analyzes the strengths and limitations of AI smart glasses across diverse healthcare application scenarios, and proposes a conceptual framework for an active health management platform based on smart glasses. The “Discussion” section further explores the practical implications of these findings for clinical practice, emphasizing the role of smart glasses in real-time health monitoring and individualized treatment planning, while also discussing current research challenges and potential future directions. The “Methods” section details the literature search and evaluation methodology, including the database retrieval strategies and the inclusion and exclusion criteria applied.

## Results

### The results of the search and selection process

A total of 863 publications were retrieved, of which 101 studies meeting the inclusion criteria were selected for systematic review. Based on the research content, the included literature was categorized into the following themes: health management (16/101), stress relief and psychological intervention (15/101), assistance in clinical surgery(23/101), tools for supporting clinical diagnosis and treatment (32/101) and telemedicine services—including telemedical education and remote diagnosis and treatment (30/101). Some of the literature covers multiple content areas. In addition, a portion of the literature focused on the ethical challenges and limitations faced by smart glasses in medical applications^42–46^, providing valuable references for future technological advancements and clinical integration.

**Fig. 2:**
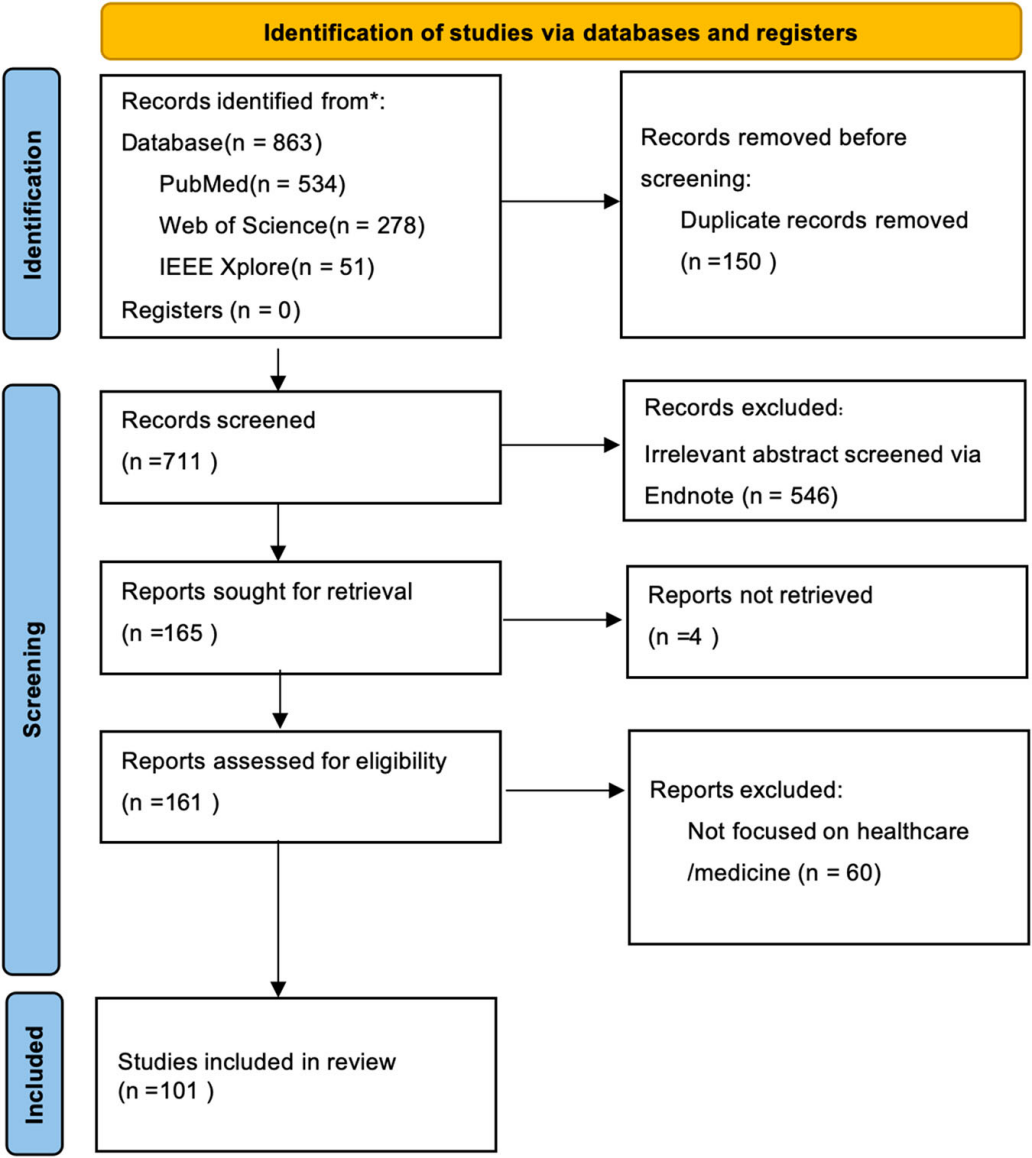
Systematic search strategy and result flowchart using the Prisma systematic review tool. This diagram illustrates the complete screening process^222^.

Figure 2 illustrates our systematic search strategy and result flowchart using the PRISMA framework, detailing the process from the identification of records through database searching to the final inclusion of studies after screening and eligibility assessment.

### Technical basis and research status of smart glasses

Smart glasses represent an advanced form of wearable computing technology designed to be worn on the head or as part of eyewear. These devices typically integrate a transparent display that overlays digital information onto the wearer’s field of vision, augmenting the physical world with real-time data^47^. The composition of smart glasses can vary widely but generally includes components such as microprocessors, sensors, cameras, connectivity modules, and user interface elements. Depending on their design and intended use, smart glasses can be classified into several categories based on their primary function or underlying technology:

Mixed reality (MR) glasses combine elements of both the real and virtual worlds to create new environments where physical and digital objects coexist and interact.

Virtual reality (VR) glasses fully immerse the user in a simulated environment, blocking out the real world entirely.

Augmented reality (AR) glasses overlay computer-generated information on top of the user’s view of the real world, enhancing the perception of reality without fully replacing it.

AI-powered glasses integrate artificial intelligence to provide context-aware assistance, predictive analytics, and personalized experiences.

Seamless integration is the standout feature of MR glasses, enabling users to interact with both real and digital objects simultaneously. This capability sets MR apart for applications requiring complex interactions, such as remote collaboration, medical training, industrial maintenance, and education. Compared to VR, MR offers a higher level of endurance and medium fashionability, and portability, making it suitable for extended use in various environments. Devices like Microsoft HoloLens2 and Meta Quest Pro are equipped with precision tracking systems that ensure stable performance without obstructing daily activities.

Total immersion characterizes VR glasses, which provide an unparalleled experience by replacing the user’s view of the physical world with a simulated one. While they score lower on portability and fashionability due to their heavy weight, VR devices excel in delivering high-resolution displays and powerful graphics processing. This makes them ideal for entertainment, gaming, education, and training scenarios where complete isolation from the external environment is beneficial. In addition, products like Meta Quest and HTC Vive prioritize sensory engagement over mobility, offering lifelike simulations that can be used for everything from architectural walkthroughs to therapeutic treatments.

Contextual enhancement without displacement is the forte of AR glasses, which overlay information onto the real world without fully replacing it. AR glasses strike a balance between functionality and wearability, with medium weight and portability that do not impede daily activities. They are well-suited for practical applications such as navigation, translation, entertainment, and photography. Devices like Microsoft HoloLens and Magic Leap offer valuable context-aware data that enhances situational awareness and decision-making. Unlike VR, AR maintains a connection to the physical world, ensuring continuous interaction while providing supplementary information.

Table 1 provides a detailed comparison of different types of smart glasses, highlighting the unique attributes and functionalities associated with each category.

**Table 1:** Comparative analysis of smart glasses categories based on functional attributes and application domains

	MR	VR	AR	AI
Fashionability	Medium	Low	Medium	High
Portability	Medium	Low	Medium	High
Weight	Medium	Heavy	Medium	Light
Application	Remote Collaboration, Medical, Industrial, Education	Entertainment, Gaming, Education, Training	Navigation, Translation, Entertainment, Photography	Healthcare, Finance, Transportation, Services, Entertainment
Endurance	High	Low	Medium	High

This table provides a comparative analysis of different categories of smart glasses based on their functional attributes and application domains, highlighting the variability in characteristics such as fashionability, portability, weight, and endurance, which collectively determine their suitability and user preferences across specific use cases.

Intelligent assistance through context-aware computing defines AI-powered glasses, which provide personalized support using machine learning algorithms. These glasses excel in sectors like healthcare, finance, transportation, services, and entertainment, thanks to their high fashionability, portability, and lightweight. Products like Ray-Ban-Meta integrate seamlessly into everyday life, offering predictive analytics and real-time recommendations based on environmental cues and historical data. The key advantage of AI-powered glasses is their ability to enhance human performance with smart, anticipatory guidance, setting them apart from other types of smart glasses that may not offer the same level of personalized assistance.

### Common smart glasses models on the market

Table 2 offers an exhaustive comparison of various smart glass models currently available on the market, with an in-depth analysis based on key characteristics. These include support for large language models, optical technology, voice recognition, image object detection, AR/VR capabilities, applications, and appearance. The information comes from the official websites of each brand.

**Table 2:** Analysis of current market smart glasses models based on key technological and functional characteristics

Brand/Model	LLM	Optics	Voice identify	Image object detection	AR/VR	Refresh rate	Resolution	Battery capacity	Sensor	OS	Application
Rokid Max AR Glasses^175^	/	Yes	Yes	/	AR	120 Hz	3840 × 1080	/	Enhanced 9-axis (gyroscope, accelerometer, magnetometer) sensor fusion scheme, distance sensor, etc.	/	Cinematic experience of giant-screen theaters, high-speed competitive gaming, multi-screen cloud-based office work, etc.
Ray-Ban Meta^176^	Yes	/	Yes	Yes	/	/	/	160 mAh	ambient light sensor, captouch sensor, etc.	/	Conversational assistant, photographing, video recording, sending messages, translation, object recognition, etc.
RayNeo Air3^177^	/	Yes	/	/	AR	120 Hz	3840 × 1080	/	Accelerometers, Gyroscope, Distance sensor, Geomagnetic sensors, etc.	/	Movie entertainment, multi-screen work, games, and office assistance, etc
Mijia smart audio glasses pilot^178^	/	/	Yes	/	/	/	/	122*2mAh	Touch Sensor, etc.	/	Audio playback, voice assistant, etc.
Xiaomi glasses camera^179^	/	Yes	Yes	Yes	AR	/	/	1020 mAh	Light Sensor, etc.	/	Periscope double camera, rapid capture, etc.
MYVU Discovery AR glasses^180^	Yes	Yes	Yes	Yes	AR	/	1280 × 480	183 mAh	Accelerometer, Gyroscope, Magnetometer, Wearable Sensor, etc.	Flyme AR	FlymeAI large language model, etc.
HUAWEI Vision Glass^181^	/	Yes	Yes	/	/	60 Hz	3840 × 1080	/	Accelerometer, Gyroscope, etc.	/	Portable assistant, Intelligent cinematic experience, etc
HUAWEI GENTLE MONSTER Eyewear II^182^	/	/	Yes	/	/	/	/	85 mAh	Vibration sensing sensor, Pressure sensing sensor, Sliding sensing sensor, etc.	/	Intelligent voice reminder and new intelligent interaction, etc.
XREAL Air 2^183^	/	Yes	/	/	AR	120 Hz	3840 × 1080	/	Gyroscope, Accelerometer, Magnetometer, etc.	/	Movie viewing experience, game interaction, and office assistance, etc.
Lucyd Glasses^184^	Yes	/	Yes	/	/	/	/	/	Touch sensor, etc.	/	Music, phone calls, voice assistants, access to ChatGPT, Information, Instruction, and Communication, etc.
Amazon Echo Frames (Gen 3)^185^	Yes	/	Yes	/	/	/	/	/	Touch Sensor, Accelerometer, etc.	/	Alexa voice assistant, music, podcasts, calls, smart notification filtering, etc.

This analysis evaluates contemporary smart glasses models, focusing on key technological features like AR, object detection, and voice interaction, as well as their applications in entertainment, assistance, and productivity. Innovations such as advanced optics and AI-driven functionalities highlight the diversity and potential of these devices. The slash (/) in the table represents that the information was not mentioned in the publicly available sources consulted by the research team.

The current market smart glasses have made significant breakthroughs in technology and functionality, but there are still many shortcomings that limit their widespread use. For example, many brands offer relatively simple functions, mainly providing basic voice reminders and broadcasting features, lacking more advanced AR/VR functions, image recognition, or intelligent interaction applications. Therefore, it is recommended to strengthen the integration of AR/VR capabilities and improve their applicability in various scenarios, especially in intelligent interaction. Additionally, the issue of battery life is still a concern. Some products have relatively small battery capacities, limiting long-term use. To improve the user experience, the battery capacity should be increased, ideally to over 400 mAh, to ensure users do not need to charge frequently during extended use. Furthermore, the display quality and resolution still need improvement. A few products offer relatively poor display quality, which is not suitable for detailed AR applications or high-definition displays. Therefore, it is recommended to increase the display resolution to at least 1920 × 1080 or higher to ensure clarity and meet AR/VR and high-quality video requirements. In terms of interactive features, many smart glasses lack voice recognition or gesture control and mainly rely on physical buttons or limited voice interaction, reducing convenience. Therefore, integrating more advanced voice assistants and gesture control features will enhance the interaction experience. Additionally, although some brands offer AR capabilities, their visual effects, refresh rates, and immersive experiences still have room for improvement. Many products lack a comprehensive AR experience, so optimizing AR display effects and increasing the refresh rate to over 120 Hz will significantly improve users’ sense of immersion and overall experience. Lastly, some brands focus too much on basic features like voice assistants, music, and calls, without fully expanding on high-end applications like AR, object recognition, and translation. Therefore, it is recommended to innovate in the area of feature expansion, adding functionalities like health monitoring, real-time translation, and object recognition to meet the needs of various user groups. In conclusion, while current smart glasses products have made breakthroughs in certain fields, to truly expand the market and enhance user experience, further improvements and optimizations are needed in areas such as battery life, display quality, interactive features, and diverse applications.

The core technology of smart glasses is intricately divided into multiple modules, encompassing both hardware and software components that facilitate the realization of intelligent functionalities. As delineated in Table 3, the hardware module serves as the fundamental basis for the functionality of smart glasses, incorporating essential components such as display systems, sensing technologies, processing units, and interaction interfaces.

**Table 3:** Details of the hardware module

Submodules	Module details
Display technology^186^	Display technologies: Micro LED displays, optical waveguides, and LCD displays are utilized to enable AR overlay functions, enhancing the visualization of digital information in the real world. Projection technology: Lasers or micromirror arrays are employed to project images directly onto the retina or spectacle lenses, creating immersive visual experiences.
Aware of the hardware^187^	Camera: Employed for environmental visual perception, facial recognition, object tracking, and gesture recognition, enabling advanced user interaction and situational awareness. Sensors: Includes technologies such as Inertial Measurement Units (IMU), GPS, and accelerometers, which facilitate motion capture and positional tracking. Microphone and speaker: Support voice command input and audio output, enabling hands-free interaction and seamless auditory communication.
Handling unit^95^	AI chips: For example, the Qualcomm Snapdragon platform, which processes visual, auditory, and multi-modal inputs in real time, enabling intelligent decision-making and adaptive functionality. Storage and battery modules: Provide essential support for data processing and ensure continuous operation, facilitating extended use and seamless performance.
Interactive devices^188^	Trackpad: Enables users to interact with the interface by swiping their fingers, providing intuitive control. Eye-tracking devices: Utilize infrared light or cameras to monitor eye movements, enabling gaze-based control and enhancing user interaction.

This hardware module analysis details the core components and functionalities of smart glasses, emphasizing display technologies like Micro LED and projection methods for immersive visuals. It highlights the role of cameras, sensors, and audio devices in enabling advanced interaction, while AI chips and storage modules ensure real-time processing and extended usability.

As delineated in Table 3, the hardware module encompasses several sophisticated submodules pivotal to advanced augmented reality systems. The display technology submodule adopts Micro LED displays, optical waveguides, and LCD displays, facilitating superior AR overlay capabilities that enhance the visualization of digital information within the physical world. Complementing this, the projection technology submodule leverages lasers or micromirror arrays to project images directly onto the retina or spectacle lenses, engendering deeply immersive visual experiences. The awareness of the hardware submodule involves cameras for environmental visual perception, facial recognition, object tracking, and gesture recognition, enabling advanced user interaction and situational awareness. Sensor’s submodule includes technologies such as Inertial Measurement Units (IMU), GPS, and accelerometers, facilitating motion capture and positional tracking. The handling unit submodule comprises AI chips, like the Qualcomm Snapdragon platform, which processes visual, auditory, and multi-modal inputs in real time, enabling intelligent decision-making and adaptive functionality. Storage and battery modules provide essential support for data processing and ensure continuous operation, facilitating extended use and seamless performance. Interactive devices submodules include trackpads for intuitive control and eye-tracking devices that utilize infrared light or cameras to monitor eye movements, enabling gaze-based control and enhancing user interaction.

As outlined in Table 4, the software and AI technology modules are crucial for the functionality of smart glasses, powering their advanced capabilities. The multi-modal data fusion submodule integrates visual, voice, and environmental data, enhancing perception and interaction through sophisticated multi-modal models (such as GPT-4). The computer vision submodule includes object detection and recognition for real-time scene analysis and SLAM technology for precise spatial positioning and navigation in augmented reality. The NLP submodule supports voice instruction processing, question answering systems, translation, and environmental semantic analysis. The AR interaction submodule utilizes AR SDKs (such as Unity, ARKit, and ARCore) to overlay and interact with virtual information. The edge computing vs. cloud computing submodule distinguishes between edge computing, which handles simple tasks on the device side to reduce latency, and cloud computing, which relies on remote servers to complete complex data calculations and multi-modal model operation.

**Table 4:** Software and AI technology modules for smart glasses

Submodules	Module details	Advantages	Disadvantages
Multi-modal data fusion	Combine visual, voice, and environmental data to integrate perception and interaction through multi-modal large models (such as GPT-4)^189,190^.	Enhances user interaction by combining multiple data streams; more accurate decision-making.	Requires significant computational resources; complexity in data synchronization.
Computer vision	Object Detection and Recognition: Recognize objects and text in a scene in real time^191^. SLAM technology: Enables precise spatial positioning and navigation in augmented reality^192^.	Real-time scene analysis; precise spatial positioning and navigation.	Performance may degrade in complex environments; requires high processing power.
NLP	Support voice instruction processing, question answering systems, translation, and environmental semantic analysis^193,194^.	Facilitates hands-free operation; improves accessibility and global connectivity.	Accuracy can be affected by ambient noise; limited understanding of context in some cases.
AR interaction	AR SDKs (such as Unity, ARKit, and ARCore) are used to overlay and interact with virtual information^195^.	Enhances user experience with immersive interactions; versatile applications.	Dependent on the robustness of AR software development kits; potential latency issues.
Edge Computing vs. Cloud Computing	Edge computing: Handles simple tasks on the device side to reduce latency^196,197^. Cloud computing: Rely on remote servers to complete complex data calculations and multi-modal model operation^197^.	Reduces latency for edge computing; supports extensive calculations via cloud.	Edge computing has limited capacity; cloud computing is dependent on internet connectivity.

This analysis outlines the software and AI technology modules integral to smart glasses, highlighting their functionalities, advantages, and limitations.

The functional modules of smart glasses are meticulously engineered to enhance user experience and expand applicability across a spectrum of use cases, including translation, navigation, health monitoring, scene recognition, and education and entertainment, as detailed in Table 5, thereby catering to a diverse array of user needs.

**Table 5:** Elaboration of functional modules for smart glasses

Submodules	Module details	Advantages	Disadvantages
Real-time translation	Instant communication in multiple languages is facilitated through speech recognition and machine translation technologies, breaking down language barriers and enhancing global connectivity^198^.	Breaks down language barriers; enhances global communication.	Translation accuracy may vary; depends heavily on the quality of input and algorithm sophistication.
Navigation & Positioning	By integrating with AR technology, smart glasses can display navigation paths or points of interest, providing users with intuitive and immersive directional assistance^199^.	Provides intuitive directional assistance; enhances user experience.	Accuracy may be affected by GPS signal strength; performance in urban canyons could be problematic.
Health monitoring	Equipped with biosensors, these glasses monitor health metrics such as heart rate and blood pressure in real time, offering users continuous health tracking and early detection of potential health issues.	Continuous health tracking; early detection of potential health issues.	Limited battery life; sensors’ accuracy might decrease over time without proper calibration.
Scene recognition	Help users understand and make decisions in complex environments, such as help visually impaired people identify obstacles, etc^200,201^.	Aids in making informed decisions; increases safety in hazardous environments.	Requires substantial training data; performance degradation in unfamiliar settings.
Education and entertainment	Smart glasses provide interactive learning experiences or immersive gaming, making education more engaging and entertainment more interactive, thus enhancing both learning outcomes and leisure activities^170^.	Engages users more effectively; promotes better learning outcomes and leisure activities.	Content creation can be resource-intensive; variability in user engagement levels.

This analysis highlights smart glasses’ functional modules, emphasizing their advantages and limitations in translation, navigation, health monitoring, scene recognition, and education.

### Application scenarios of smart glasses

The core applications of smart glasses encompass visual assistance, smart home control, navigation and positioning, information prompts and reminders, social interaction support, health monitoring, and education and training. Leveraging advanced AI technology and sensor integration, smart glasses offer personalized life support through augmented reality (AR), voice interaction, and other technical capabilities. For example, for visually impaired users, smart glasses can integrate object recognition, real-time obstacle detection, and path planning features, significantly enhancing travel safety and overall convenience^48,49^.

In the realm of smart homes, smart glasses serve as central control hubs, managing functions such as lighting, temperature, and security systems through voice or visual commands. Building on this capability, the HUAWEI Smart Glasses 2 further extends its functionality by offering application reminders, including weather updates, schedule summaries, and health monitoring, with features like cervical spine fatigue detection^50^.

Additionally, there are sports-oriented smart glasses designed for fitness enthusiasts, capable of recording exercise data such as steps, heart rate, and GPS routes, providing valuable scientific insights to support training and fitness goals^51^. In the field of education, smart glasses offer an AR-enhanced learning experience, featuring functions such as translation and recording, supported by visual assistance and interactive learning modules to enhance both learning efficiency and engagement.

**Fig. 3:**
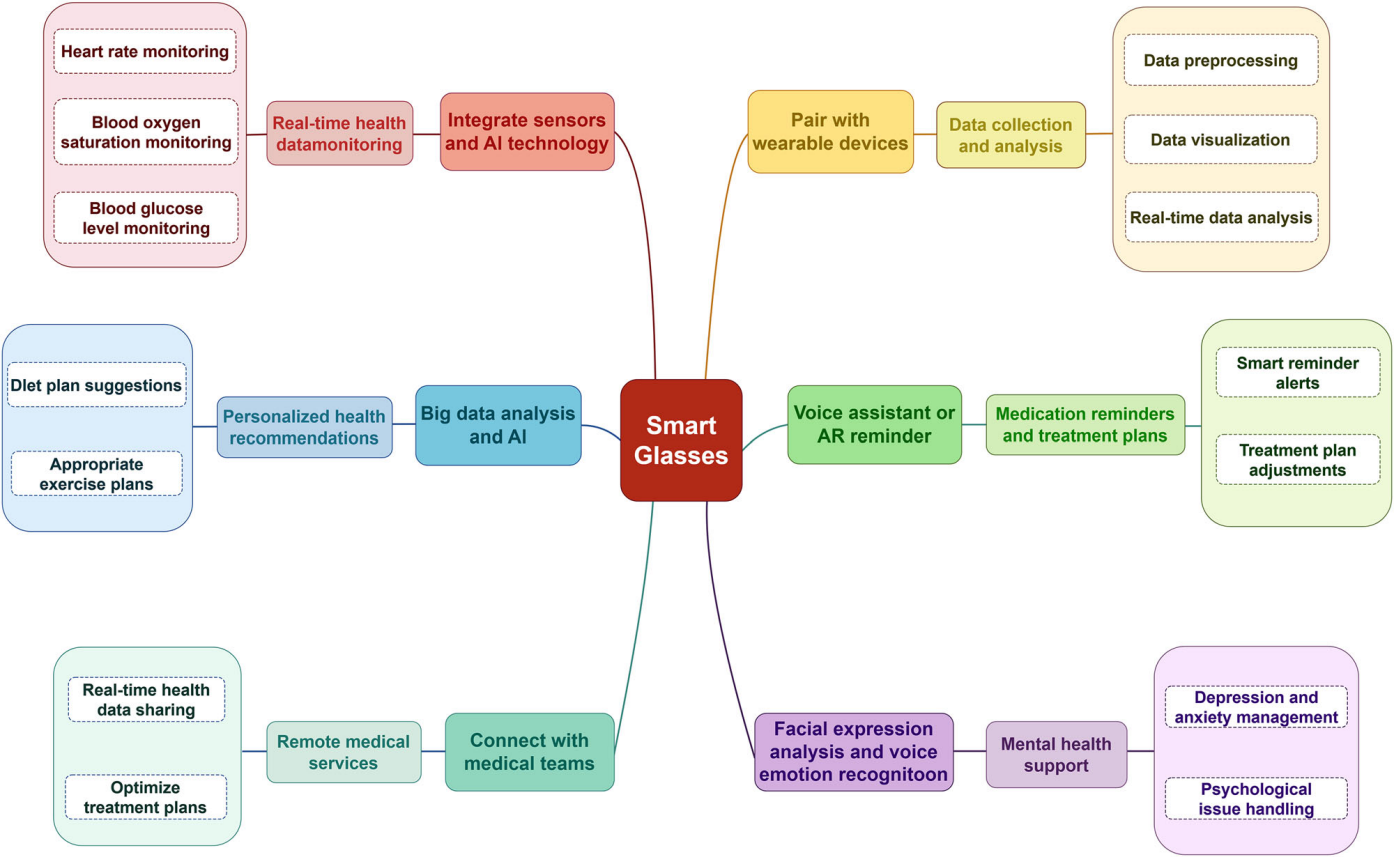
Advanced applications for smart glasses in healthcare monitoring. Smart glasses integrate sensors and AI technology, pair with wearable devices, and utilize big data analytics to enable real-time health monitoring, personalized health recommendations, telemedicine services, and mental health support (By Figdraw).

Healthcare represents a key development focus for smart glasses, with core technologies encompassing large multi-modal models, high-precision sensors, and real-time data analysis. In the realm of chronic disease management, a study by Guan et al.^52^ highlights the pivotal role of AI in the prevention and management of diabetes, a field with significant development potential, smart glasses can leverage integrated multi-modal large models and AI algorithms to monitor the health trends of patients with chronic diseases. Current trends focus on incorporating smart sensors into wearable devices, enabling continuous health monitoring within the user’s natural environment^53^. Figure 3 illustrates the various application possibilities of smart glasses.

By incorporating optical sensors, infrared cameras, and edge computing modules, smart glasses can collect and analyze users’ health data, such as heart rate, blood sugar levels, and blood pressure, in real time, offering precise health monitoring and management for patients. For example, Microsoft’s blood pressure monitoring smart glasses utilize optical technology to quickly and accurately record blood pressure metrics^54^. In the domain of blood glucose monitoring, smart glasses are still under research and development. However, several projects have already demonstrated the feasibility of this technology. Park et al. introduced the development of a wireless smart contact lens glucose biosensor, showcasing its ability to monitor glucose levels as a non-invasive alternative to traditional blood glucose measurements, highlighting the potential of smart contact lenses in non-invasive glucose monitoring^55^. Emteq Labs’ smart glasses, Sense, can monitor health and capture data at a rate of 6000 times per second^56^. VR glasses have been applied intraoperatively to monitor patients’ emotional states and deliver interventions—including guided meditation, relaxation techniques, and distraction strategies—to effectively alleviate anxiety, stress, and pain during surgical procedures^57–63^. Based on the current developments of smart glasses in the field of mental health, this study proposes future application scenarios for mental health management using smart glasses, as outlined in Table 6.

**Table 6:** Application and principle of smart glasses in mental health management and virtual follow-up health services

	Mental health management	Virtual follow-up and health management
Function	Patients often experience mood swings or stress, and smart glasses can monitor the patient’s emotional state and provide stress management, meditation or relaxation training^58,59^.	The voice assistant built into the smart glasses can provide psychological support, such as voice comfort, meditation exercises, or psychological counseling.
Apply	Smart glasses integrated with mental health monitoring and intervention capabilities allow individuals experiencing depression or anxiety to continuously track and regulate their emotional states, thereby facilitating the mitigation of negative affective symptoms^106,202–205^.	Patients can self-regulate themselves through an app on their glasses when they feel anxious.
Principle	The glasses use facial expression recognition, voice analysis, and physiological indicators such as heart rate changes to assess the patient’s emotional state and recommend interventions^206–208^.	Through emotion recognition technology, glasses can capture a patient’s mood changes and provide psychological intervention or relaxation techniques through a voice or visual interface.

This analysis explores smart glasses’ role in mental health management and virtual follow-up services in the future, utilizing emotion recognition, voice analysis, and physiological monitoring to provide stress management, psychological support, and self-regulation tools for users experiencing anxiety or depression.

As data collection improves and technology advances, smart glasses are poised to play a pivotal role in personalized health management and intervention. By leveraging multi-modal data analysis, these glasses can provide patients with tailored health recommendations on diet, exercise, and more^64–67^. Additionally, they can remind patients to take their medications on time and assess the effectiveness of these treatments. This personalized health management service is particularly beneficial for individuals with chronic diseases, as it offers real-time feedback, empowering patients to make timely adjustments to their lifestyle or medication regimens, thereby enhancing their daily lives and overall well-being^68–72^. Table 7 provides a detailed description of personalized health advice and reminders, as well as medication management and reminders, based on the capabilities of smart glasses.

**Table 7:** Personalized health advice and reminders, medication management and reminders based on smart glasses

	Personalized health recommendations and reminders	Medication management and reminders
Function	Based on real-time health data, Smart glasses analyze a patient’s condition and provide personalized health advice, dietary advice, or exercise guidance through a voice or visual interface.	Smart glasses can remind patients to take their medications on time and monitor the effects of their medications based on their medication history, medical records, and disease status.
Apply	For people with high blood pressure, glasses can provide reminders to take medications and provide dietary or exercise advice to help control blood pressure^209^.	For patients with chronic diseases, especially those who need to take medication for a long time, glasses can help patients take their medications on time and alert them to abnormal conditions^209^.
Principle	The AI algorithm analyzes the user’s health data, combined with the patient’s personal medical history and health goals, to generate a personalized health plan^67^.	The glasses’ built-in schedule reminder function, combined with an intelligent voice assistant, provides medication reminders at a user-defined time and monitors medication progression^209^.

This analysis examines smart glasses’ capabilities in delivering personalized health advice and medication management in the future. By leveraging AI algorithms and real-time health data, they provide tailored recommendations for diet, exercise, and medication adherence, while monitoring patient progress and alerting on abnormalities to enhance chronic disease management.

Smart glasses can assist clinicians in capturing, recording, and storing key findings during consultations, eliminating the need for manual data entry or the use of a scribe^73–77^. They also facilitate electronic medical record management, enabling direct conversion of records to electronic format, which significantly reduces the time spent transferring data from physical files^78–81^. With the integration of AI technology, clinicians can analyze rapid test results and gain insights to optimize patient care. Additionally, smart glasses serve as valuable tools for surgeons during procedures^46,82–85^. Smart glasses have been applied to different types of clinical procedures^64,86–92^. Following the release of Google Glass, Dr. Phil Haslam and Dr. Sebastian Mafeld demonstrated its potential in interventional radiology. They showcased how Google Glass could assist in liver biopsies and fistuloplasties^93^, potentially enhancing patient safety, improving operator comfort, and increasing surgical efficiency.

As exemplified by the smart glasses developed by Vuzix specifically for telemedicine applications^94^. These glasses can bridge the distance between doctors and patients through voice connectivity, which enables caregivers to instantly share medical expertise with healthcare professionals worldwide, offering life-saving guidance from any location^95–101^. They facilitate real-time exchange of expert medical feedback without compromising patient care, while also providing surgeons with immediate input to reduce errors and enhance surgical precision through AR technology^102–104^. For patients in remote areas, smart glasses offer doctors the ability to monitor patients’ conditions in real time through remote video calls and data sharing, bringing convenient healthcare services directly to those in need^105^. This model helps address the challenges of unequal distribution of medical resources and significantly enhances the efficiency and accuracy of patient follow-up. Table 8 showcases the role of smart glasses in telemedicine.

**Table 8:** Utilization of smart glasses in telemedicine services for enhanced remote health management and virtual follow-up

	Telehealth support	Virtual follow-up and health management
Function	Smart glasses can support real-time communication between patients and doctors through video calls, remote diagnosis, real-time data sharing and other functions^154,210–217^.	Smart glasses can be used as a health management platform to help doctors remotely monitor and follow up on their condition.
Apply	Through the glasses’ camera, the doctor can see the patient’s symptoms and make recommendations in real time, especially for patients living in remote areas^96,218,219^.	Patients wear glasses every day, perform health data collection and connect with the medical system, and doctors track changes in patients’ conditions through data analysis^69^.
Principle	Smart glasses can transmit patients’ health data and symptom images in real time and provide them to remote doctors in combination with AI analysis results^154,216,220,221^.	The built-in data synchronization of smart glasses relates to the cloud platform, and doctors can obtain patients’ health data in real time and provide personalized guidance and intervention.

This analysis highlights the role of smart glasses in telemedicine and virtual follow-up, enabling real-time patient-doctor communication, remote diagnosis, and health data sharing. Integrated with AI, they facilitate remote monitoring, symptom analysis, and personalized interventions, enhancing accessibility and continuity of care, particularly for remote or chronic disease patients.

With the continuous advancement of sensor technology and AI algorithms, smart glasses will gradually become miniaturized and adapt to more medical scenarios^106,107^. The combination of these technologies will not only improve the quality of medical care, but also optimize the allocation of medical resources and provide personalized health management services for more people^108^.

The industrial applications of smart glasses, leveraging AR, Internet of Things (IoT) connectivity, AI analytics, and high-precision vision and motion sensing technologies, have the potential to significantly enhance productivity, safety, and product quality. The following Table 9 outlines specific application scenarios.

**Table 9:** Industrial applications of smart glasses leveraging AR, IoT, AI analytics, and high-precision sensing

	Apply	Case	Principle
Security monitoring and emergency	Sensors built into the glasses monitor workers’ environmental conditions (e.g., gas leaks, temperature anomalies) and provide real-time warnings^30^.	Mining company uses smart glasses to improve the safety of underground operations^30^.	Sensors and AI algorithms are used to monitor environmental data and determine potential risks in combination with worker status.
Production assembly and quality control	Smart glasses provide workers with dynamic guidance on assembly steps or automatically detect product defects during quality control^31^.	BMW uses smart glasses in production line assembly to reduce the rate of operator errors^31^.	Smart glasses provide workers with dynamic guidance on assembly steps or automatically detect product defects during quality control.
Logistics & Warehouse Management	In the warehouse, smart glasses can guide workers to quickly find the location of items through AR technology to improve sorting efficiency^32^.	Amazon uses smart glasses for shipment location and real-time inventory management^32^.	Through barcode scanning, GPS positioning, and AR navigation, the glasses show the specific storage location of the goods and update the system in real time.
Remote collaboration and training	Engineers can share first-person perspectives through smart glasses, and experts can remotely guide them to solve complex problems. It is also used to train new employees^33^.	Boeing uses smart glasses in aerospace assembly training to significantly reduce learning time^33^.	Smart glasses transmit video and voice over a high-speed network, combined with AR annotation capabilities to achieve efficient collaboration.
Equipment maintenance and fault diagnosis	The smart glasses provide real-time guidance to technicians by overlaying equipment operation manuals and fault information through AR technology^34^.	Daimler and GE use AR glasses to reduce equipment downtime and improve maintenance efficiency^34^.	The glasses capture real-time images of the device through the camera, and combined with AI analysis, identify the problem parts, and superimpose the maintenance plan.

This table highlights industrial applications of smart glasses in security, production, logistics, collaboration, and maintenance, leveraging AR, IoT, and AI for enhanced efficiency and safety.

### The role and challenges of smart glasses in health management

Under the concept of active health, there is growing emphasis on maintaining a healthy diet and ensuring food quality, with the detection and analysis of food nutrients becoming a major research focus. The proper intake of nutrients such as proteins, fats, carbohydrates, vitamins, and minerals is essential for human health^109^. Consequently, the development of efficient and precise nutrient detection technologies is crucial for formulating evidence-based dietary plans and upholding food safety standards. Traditional chemical analysis methods, however, are often time-consuming and complex, frequently requiring destructive sampling that limits their practical application^110^.

Recent advancements in computer vision and deep learning have revolutionized non-destructive nutrient assessment. These technologies excel in automatic feature extraction, accurate classification, and end-to-end learning, positioning them as indispensable tools for food image recognition and nutritional evaluation. For example, the NutriNet system utilizes convolutional neural networks to achieve high classification accuracy but has limitations when processing multi-component images^111^. The MResNet-50 model, enhanced by natural language processing (NLP), enables automatic recipe extraction, addressing intra-class variability in food images, yet demonstrates limited generalization capabilities for unseen categories^112^. Wang et al.’s model integrates EfficientNet^113^, Swin Transformer, and Feature Pyramid Network (FPN) to adapt to complex scenarios, though it requires further development for detailed component identification in traditional Chinese dishes^113–116^.

The Im2Calories app exemplifies this progress by combining segmentation and classification techniques to evaluate meals with an accuracy rate of 76%, providing a robust solution for fine-grained differentiation^117^. Liu et al.’s multi-dish recognition model employs EfficientDec to enhance the accuracy of dietary intake reporting, although it necessitates frequent dataset updates to account for seasonal variations^118^. The ChinaMarketFood109 database has been instrumental in training Inception V3, improving image classification accuracy; however, there remains room for improvement in estimating nutrient content. Emerging models like DPF-Nutrition leverage monocular images along with depth prediction modules to estimate food nutrition, though they encounter limitations when processing stacked images^119^. The RGB-D feature fusion network integrates color and depth information, enhancing multi-modal learning capabilities and offering solutions for occlusion management and the recognition of complex scenes^120^. From food image recognition to comprehensive nutritional assessment, deep learning and multi-modal technologies demonstrate significant potential. However, challenges related to adaptability in complex scenarios, model generalizability, and computational costs must be addressed.

**Fig. 4:**
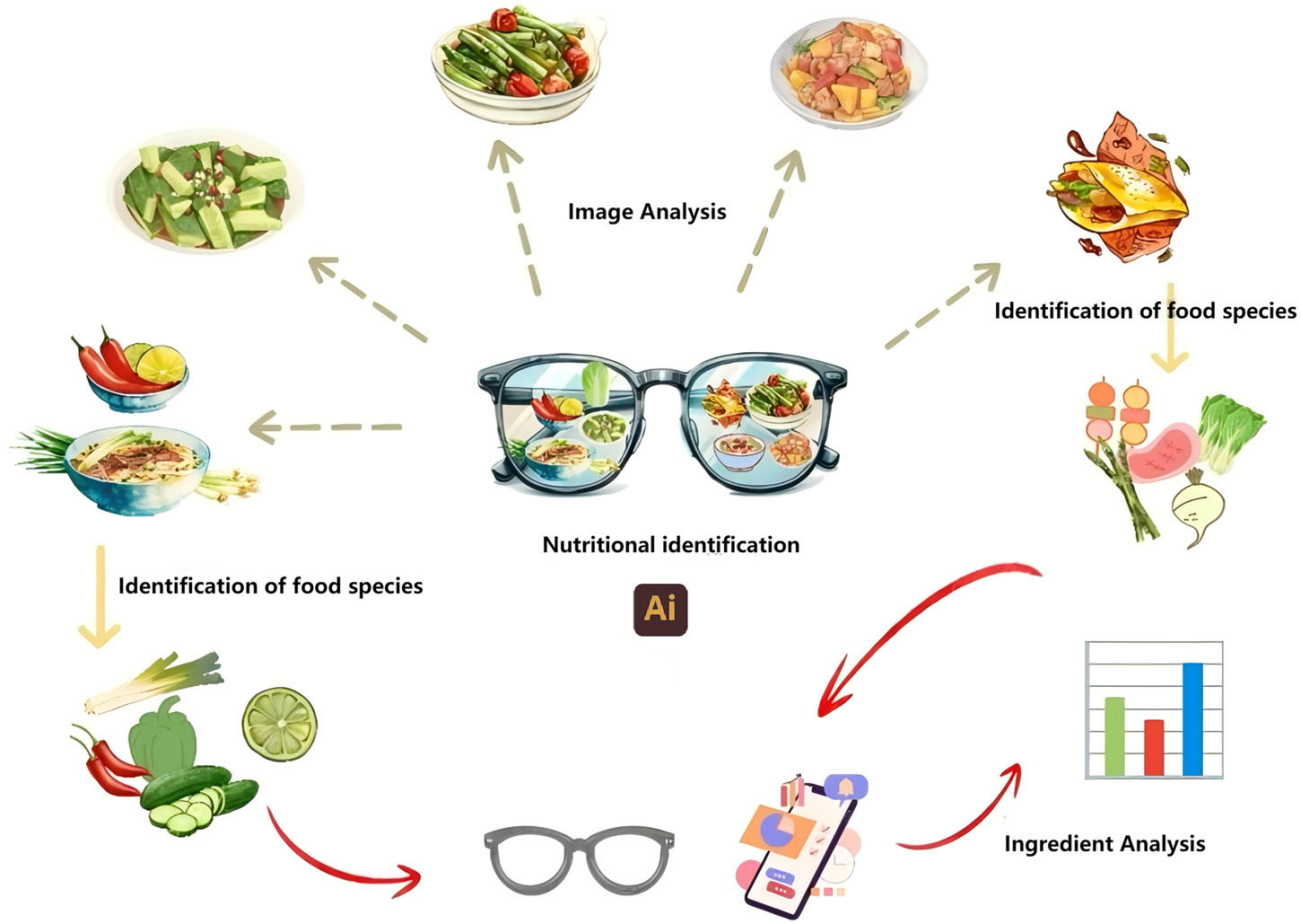
A smart glasses-based application framework for food nutrition recognition. Initially, real-time food identification is facilitated through the smart glasses’ integrated camera and advanced image recognition capabilities, which swiftly scan and analyze the visual characteristics of food. The AI algorithm subsequently cross-references the captured image data with an extensive nutritional database, thereby providing users with detailed insights into the food’s composition, encompassing calories, proteins, fats, sugars, and other essential nutrients. Nutritional information is presented in an intuitive and engaging format, equipping users with valuable and actionable dietary knowledge.

Figure 4 depicts the workflow of smart glasses in the realm of food nutrition recognition. This application is primarily designed to offer real-time food identification^121^, comprehensive nutritional analysis, and tailored health recommendations by integrating state-of-the-art computer vision^122^, AI, and AR technologies. Initially, real-time food identification is facilitated through the smart glasses’ integrated camera and advanced image recognition capabilities, which swiftly scan and analyze the visual characteristics of food. The AI algorithm subsequently cross-references the captured image data with an extensive nutritional database, thereby providing users with detailed insights into the food’s composition, encompassing calories, proteins, fats, sugars, and other essential nutrients. Nutritional information is presented in an intuitive and engaging format, equipping users with valuable and actionable dietary knowledge. For instance, ChatDiet^123^ realizes personalized nutrition-oriented food recommendations through an LLM-augmented framework. It integrates individual and population models. The individual model employs causal discovery and reasoning techniques to evaluate the nutritional effects on specific users, while the population model provides generalized nutritional information about food. The coordinator transmits the outputs of both models to the LLM, thereby offering customized food recommendations. The effectiveness of its food recommendation test reaches 92%.

Utilizing AI technology, smart glasses seamlessly superimpose real-time nutritional information onto the user’s field of vision. By simply directing their gaze at food, users are automatically presented with relevant nutrient data and health recommendations. This functionality empowers users to conduct swift nutritional assessments prior to consumption, thereby facilitating healthier decision-making. Moreover, by incorporating user-specific health data—such as weight, age, activity level, and health goals—the smart glasses can deliver personalized dietary suggestions based on their real-time food recognition capabilities. For example, if the system detects the consumption of high-sugar food, the glasses may prompt the user to monitor their sugar intake or suggest healthier alternatives. Furthermore, the glasses can integrate with the user’s broader health management ecosystem, such as a smartwatch or health app, to provide a more holistic health assessment. By continuously monitoring eating habits and physical activity, these integrated devices offer long-term solutions for personalized health management.

### Integrated active health management platform combining smart glasses and health IoT devices

**Fig. 5:**
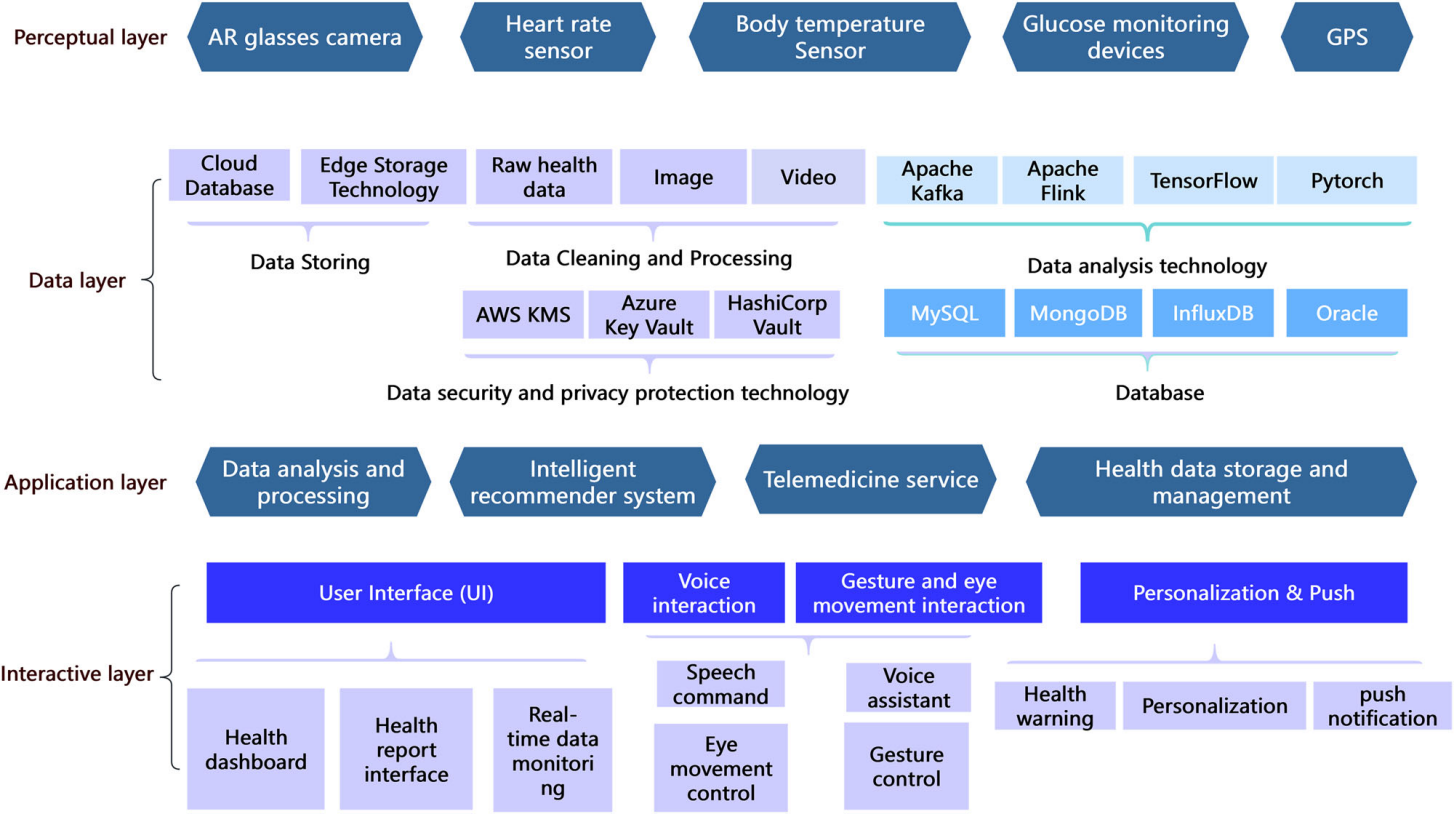
Platform architecture and functional module. The basic architecture of the platform is composed of the perceptual layer, data layer, application layer, and interactive layer.

The proposed platform architecture for AI-powered smart glasses is designed to support proactive health management through a multi-layered approach, integrating advanced technologies to ensure seamless functionality as shown in Fig. 5. The architecture consists of four hierarchical layers: the perceptual layer, data layer, application layer, and interactive layer. Each layer is strategically designed to leverage the latest advancements in technology, ensuring a cohesive system that supports real-time health monitoring and personalized health management.

The perceptual layer comprises a suite of hardware sensors, including AR glasses cameras, heart rate sensors, body temperature sensors, glucose monitoring devices, GPS modules, and other wearable sensors. These components are responsible for real-time data acquisition, enabling comprehensive physiological and environmental monitoring. For example, wearable hydrogel-based health monitoring systems can provide real-time monitoring of health indicators such as glucose, uric acid, lactose, heart rate, blood pressure, and temperature. Additionally, flexible self-powered bioelectronics (FSPB) can dynamically monitor physiological signals, revealing real-time health abnormalities and providing timely, precise treatments^124^.

The data layer facilitates the processing, storage, and management of the raw data collected by the perceptual layer. Core modules include: Data storage, utilization of cloud databases, and edge storage technologies to store diverse data formats such as images, videos, and health metrics. Cloud databases collect, deliver, replicate, and push data to the edge using hybrid cloud concepts, ensuring efficient data management^125^. Data cleaning and processing, technologies like Apache Kafka, Apache Flink, and TensorFlow are employed for efficient data preprocessing and integration. Data analysis and security, advanced analytical frameworks (e.g., MySQL, MongoDB, InfluxDB) combined with encryption tools such as AWS KMS and Azure Key Vault ensure robust data analysis and compliance with privacy standards.

The application layer encapsulates the core functionalities of the platform, focusing on health management and user engagement: Data analysis and processing, algorithms for advanced data interpretation, including health trend predictions and anomaly detection. Deep learning algorithms, which have achieved great success in image processing and speech recognition, are expected to open new depths for health monitoring systems. Intelligent recommender system, personalization of health interventions through AI-driven insights. Telemedicine services facilitate remote consultation and real-time diagnosis, bridging gaps in healthcare accessibility. Health Data Management ensures organized and secure storage of user health records for continuous monitoring and evaluation.

The interactive layer is designed to enhance user experience through multi-modal interaction mechanisms. It includes: User interface (UI), features such as a health dashboard, real-time data monitoring, and health report interfaces for intuitive visualization. Interaction modules, speech command, voice assistant, gesture control, and eye-movement interaction for hands-free operation and accessibility. Multi-modal interaction mechanisms, such as those involving LLMs, can enhance text processing abilities and provide more intuitive user experiences. Personalization and push notifications deliver customized health warnings, insights, and recommendations to the user in real time.

This multi-layered design creates a cohesive system that seamlessly integrates hardware capabilities with advanced software functionalities. It facilitates real-time health monitoring, personalized health management, and enhanced interaction for a wide range of users. The platform’s modular structure ensures scalability, adaptability, and robustness, allowing it to meet the evolving needs of next-generation wearable health technologies.

To enhance the data processing capacity and analysis accuracy of the active health management platform integrated with smart glasses and IoT devices, a multi-dimensional approach is essential. This approach involves strengthening data quality, leveraging advanced data processing techniques, and optimizing the data storage and analysis architecture.

The platform needs to ensure the integration of data from smart glasses, IoT devices (e.g., smart bracelets, smart scales, blood pressure monitors), and other health-related devices. This data should encompass physiological signals, signs, behaviors, and environmental factors to ensure multi-dimensional and diversified inputs. High-precision sensors are critical for real-time data collection, as they reduce data errors and enhance health monitoring reliability.

Regarding data storage and processing architecture, we can leverage edge computing to shift preliminary data analysis and filtering from the cloud to the device side (e.g., smart glasses or smart devices). This will reduce data transmission delay and bandwidth requirements, making real-time health monitoring more efficient. Edge computing will enhance data processing timeliness, while distributed database technology can store large volumes of health data. By scaling out, the platform can efficiently process massive amounts of data while maintaining stability. Additionally, a cloud-based big data analysis framework will process complex health datasets. Using distributed computing and storage ensures the platform can handle various data types at scale and generate real-time analysis reports based on user needs.

To improve the accuracy of data analysis and modeling, machine learning methods such as deep learning (e.g., neural networks)^126^, reinforcement learning^127^, and support vector machines (SVMs)^128^ are employed to analyze and predict users’ health data. Continuous optimization and training of these models enhance the accuracy of the analysis. Furthermore, personalized recommendation algorithms can be developed based on the user’s health history, physical characteristics, and behavioral data. These algorithms provide precise health recommendations tailored to the user’s specific situation, such as chronic medical history and genetic characteristics.

The integration of traditional Chinese and Western medicine theories is achieved by combining the constitution identification of traditional Chinese medicine with the modern medical data of Western medicine to build a hybrid model. This approach improves the predictive ability of users’ health status and the accuracy of dietary recommendations. Natural Language Processing (NLP) technology is used to analyze TCM literature^129^ and Western medicine research, integrating these theories to make personalized health recommendations. By implementing these multi-dimensional measures, the platform not only enhances the accuracy and reliability of health data analysis but also provides users with more personalized and effective health management services. This approach aligns with the latest advancements in technology, ensuring a cohesive system that supports real-time health monitoring and personalized health management.

The synthesis of smart glasses with Internet of Things (IoT) medical devices represents a pivotal advancement in the realm of active health management platforms. Central to this integration is the establishment of robust, seamless connectivity and interoperable data exchange protocols that facilitate real-time physiological monitoring. As a core wearable technology, smart glasses are envisioned to be outfitted with a comprehensive suite of advanced biosensors, including but not limited to heart rate monitors, pulse oximeters, thermometers, gait analyzers, and accelerometers. These sensors continuously capture granular biometric data from the user.

Supplementing the capabilities of smart glasses, IoT-enabled medical devices such as ambulatory blood pressure monitors, continuous glucose monitors, and bioimpedance scales provide additional critical health parameters, thereby enriching the dataset with metrics like arterial pressure, glycemic levels, and anthropometric measures. This synergistic integration ensures the integrity, comprehensiveness, and precision of the collected health information, offering a panoramic overview of the individual’s wellbeing^130^.

**Fig. 6:**
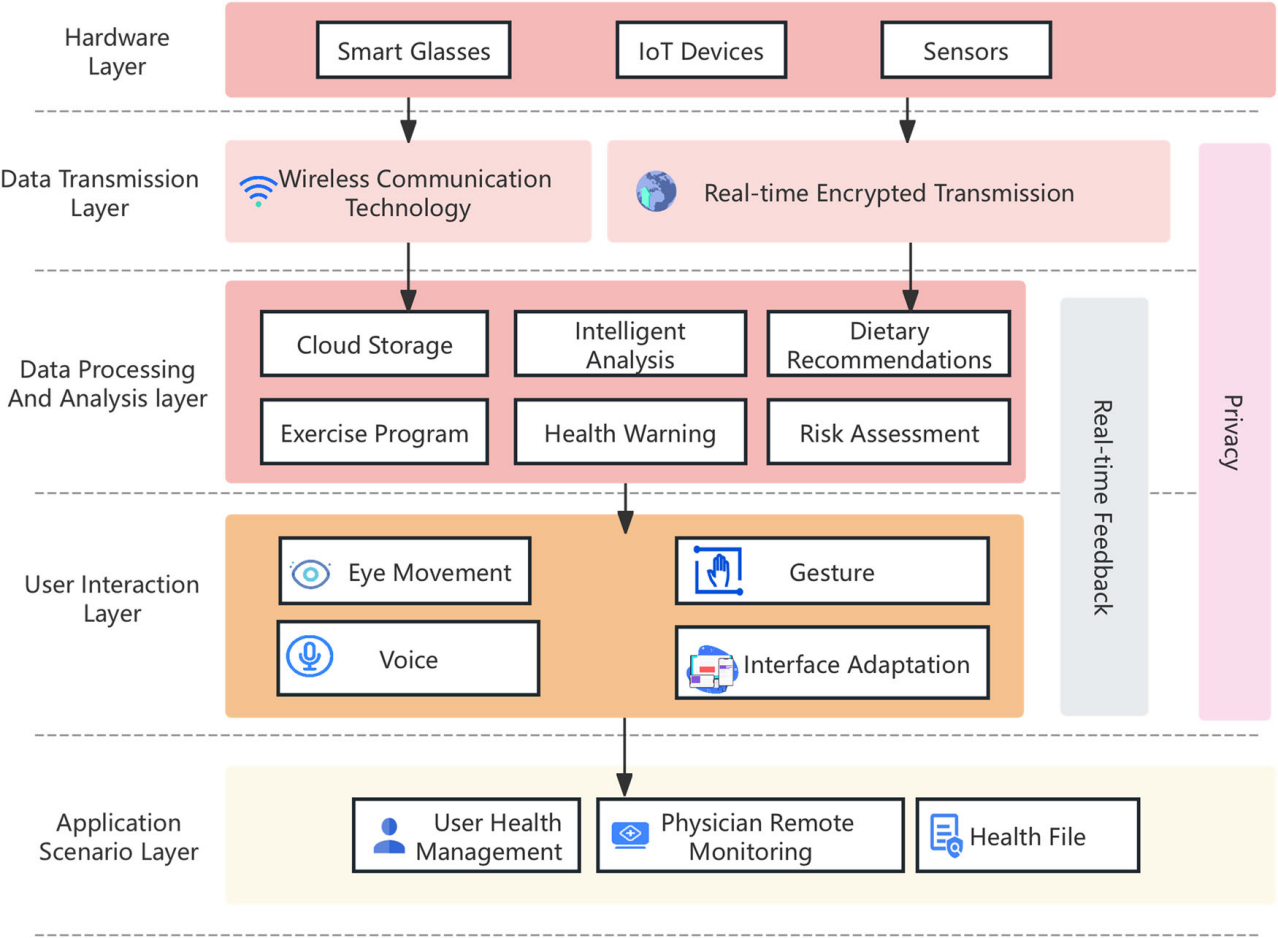
Design of integrated solutions for smart glasses and IoT devices. The intelligent health system architecture encompasses the hardware layer (including smart glasses, IoT devices, sensors), the data transmission layer (wireless communication and real-time encrypted transmission), the data processing and analysis layer (cloud storage, intelligent analysis, etc.), the user interaction layer (interaction methods such as eye movement and gestures), and the application scenario layer (such as user health management), while also taking into account privacy and real-time feedback.

Figure 6 illustrates the design of integrated solutions for smart glasses and IoT devices. The intelligent health system architecture encompasses the hardware layer (including smart glasses, IoT devices, sensors), the data transmission layer (wireless communication and real-time encrypted transmission), the data processing and analysis layer (cloud storage, intelligent analysis, etc.), the user interaction layer (interaction methods such as eye movement and gestures), and the application scenario layer (such as user health management), while also taking into account privacy and real-time feedback.

In terms of data transmission, both smart glasses and IoT devices should employ low-power wireless communication standards—such as Bluetooth Low Energy (BLE)^131^, IEEE 802.11 Wi-Fi, or ZigBee^132^—to ensure real-time data synchronization. Smart glasses aggregate biometric data from daily activities and transmit it via secure, encrypted channels over a wireless network to a cloud-based platform for storage and processing^133^. Data security and privacy are paramount; therefore, all transmissions comply with stringent encryption protocols and adhere to pertinent data protection regulations and industry standards.

For analysis and processing, the cloud platform will consolidate multi-modal datasets from smart glasses and IoT devices, harnessing machine learning algorithms and AI for sophisticated analytics. The system will perform continuous health status surveillance, anomaly detection, and trigger alerts or recommendations when deviations from baseline health metrics are observed. For instance, upon detecting tachycardia or bradycardia, the system would promptly notify the user^134^ and advise appropriate actions, such as resting or seeking medical consultation. Moreover, the platform will generate personalized health management strategies based on each user’s medical history and lifestyle factors, providing tailored services like physical activity guidance and nutritional counseling^135^.

The multi-modal interaction design of smart glasses enhances user engagement with the health management platform. Leveraging NLP for voice commands, eye-tracking^49,136^ for interface navigation, and capacitive touchscreens for manual input, the glasses dynamically adapt their visual displays according to the user’s health indicators, offering real-time monitoring and relevant health advisories.

At the application level, the convergence of smart glasses and IoT devices significantly benefits both end-users and healthcare practitioners. Users gain tools for proactive health management, while clinicians can remotely monitor patient health and provide timely interventions^137^. Through the integrated platform, physicians can access remote patient monitoring (RPM)^138^ data, evaluate conditions, assess therapeutic efficacy, and devise personalized care pathways, thus enhancing telemedicine capabilities. Furthermore, the platform facilitates the compilation of detailed, longitudinal health records, archiving all user health data for future reference. Such a repository plays an indispensable role in ongoing health maintenance and predictive analytics for disease prevention.

**Fig. 7:**
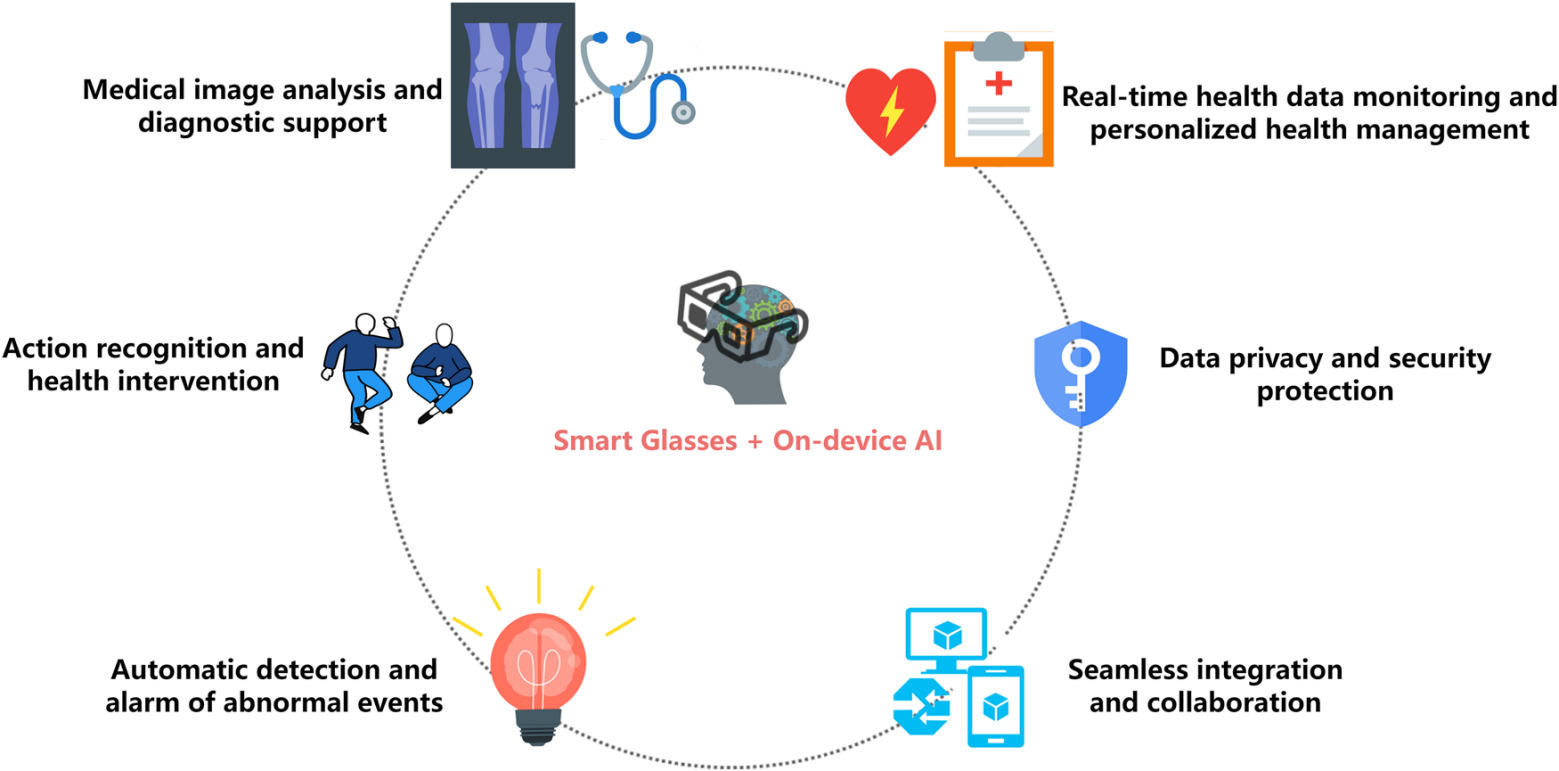
Application scenarios of on-device AI technology. Application scenarios rely on smart glasses to achieve diversified application forms.

Building an active health management platform based on the integration of smart glasses and IoT medical devices involves the integration and collaboration of smart glasses with a variety of IoT devices (such as health monitoring devices and medical sensors) through device-side AI technology. The application of edge AI technology can ensure that data is processed locally on the device (i.e., smart glasses or other IoT devices) in real time, reducing latency, improving response speed, and data security, while reducing the dependence on cloud processing^139^. Figure 7 shows different application scenarios of on-device AI technology.

For the application of real-time health data monitoring and personalized health management, smart glasses leverage built-in sensors and IoT devices (such as blood glucose meters, heart rate monitors, and blood pressure monitors) to collect real-time health data, enabling continuous health monitoring. The device-side processing capabilities of smart glasses allow for the analysis of physiological data (e.g., heart rate, blood glucose, and body temperature) directly on the device. With embedded AI chips, the glasses can analyze this data in real time and provide immediate feedback, even identifying health abnormalities (such as high blood sugar or abnormal heart rate) and issuing automatic warnings. This minimizes reliance on cloud services, ensuring real-time monitoring and improving the timeliness and accuracy of health management. Additionally, by integrating with IoT devices like exercise trackers and sleep monitors, the smart glasses offer personalized health recommendations. For instance, based on daily activity and health data, the system may suggest increasing physical activity, adjusting diet, or improving sleep quality. Through localized AI models, the glasses analyze users’ health data and create tailored intervention strategies. As users’ health data evolves, the AI system adapts the recommendations, ensuring continuous, personalized health management that is both network-independent and responsive to real-time needs. And for action recognition and health interventions, smart glasses, in combination with motion sensors (such as IoT smart bracelets), can monitor the user’s movements in real time and detect potential health-risk behaviors, such as prolonged sitting or improper exercise. By leveraging the visual and motion recognition capabilities of the smart glasses, along with motion data from IoT devices, the integrated AI system can evaluate the user’s activity posture and behavior (e.g., sitting or walking posture) in real time. The system provides corrective guidance instantly, reducing data latency with local processing and feedback. This ensures real-time monitoring and prompt, actionable guidance for improving posture and preventing health risks.

Aiming at the application of medical image analysis and diagnostic support, smart glasses can be seamlessly integrated with IoT devices (such as smart thermometers and blood pressure monitors) and medical imaging equipment (like portable ultrasound and X-ray machines) to assist healthcare professionals in diagnosing conditions. Through computer vision technology, smart glasses analyze real-time image data of the user, combining it with health data from IoT devices to facilitate quicker diagnoses. For example, by visually assessing the user’s face and physical signs, smart glasses can detect potential health issues (such as paleness or eye abnormalities) and provide real-time recommendations to doctors. This integration minimizes data transfer needs and cloud dependencies, ensuring faster diagnostic support.

For automatic detection and alarming of abnormal events, IoT devices integrated with smart glasses can promptly detect health emergencies (such as falls or seizures) and alert medical personnel or family members. By leveraging the sensors and image processing capabilities of smart glasses, along with IoT devices (like smart bracelets and environmental sensors), the system continuously monitors the user’s physiological state. In the event of an emergency, such as a fall or significant heart rate fluctuations, the AI system responds immediately by sending an alarm signal to ensure timely assistance. On-device AI ensures rapid response and processing, reducing dependence on cloud services and enhancing response speed. Seamless integration and collaboration can also be achieved. Smart glasses not only integrate with individual IoT devices but also seamlessly collaborate with multiple devices to enhance data sharing and the comprehensiveness of health management. Through on-device AI technology, smart glasses can interact with various IoT devices, such as smart bracelets, smart home systems, and environmental monitoring devices, to exchange data and provide holistic health management. For instance, based on the user’s health data, smart glasses can automatically adjust the smart home environment—such as temperature, humidity, and air quality—to optimize comfort and well-being.

By deploying edge computing on smart glasses and IoT devices, data processing tasks are moved to edge nodes locally or close to the device. This reduces the latency of data transmission and enables real-time data synchronization and processing between devices, ensuring consistency and accuracy of information. Then the real-time data synchronization protocol is adopted, such as Message Queues Telemetry Transport (MQTT)^140^, WebSocket^141^, to ensure efficient and stable data transmission between devices. These protocols are designed for low-latency and efficient communication to ensure synchronization and data consistency between devices, especially in IoT environments. And use data consistency algorithms and verification mechanisms, such as Cyclic Codes for Error Detection, Unix timestamp, to ensure that the transferred data is not lost or corrupted. Even if there is a delay or disconnection in communication between devices, the system ensures data integrity and consistency. A distributed architecture can be designed to ensure seamless connection between the local data processing of smart glasses and IoT devices and cloud systems^142^. The device can process the data locally and upload the results to the cloud for further analysis or storage, ensuring data synchronization and consistency. Set up the device to continue to collect data when it is offline and compensate for the data when the network is restored. The system can ensure consistency between offline and online data through mechanisms such as timestamping and versioning, avoiding data loss or duplication. This ensures consistency and accuracy of data between devices. The combination of these technologies can improve the efficiency and stability of the active health management platform.

## Discussion

In recent years, the rapid development of wearable technology, especially smart glasses, has brought significant innovations to the healthcare field, particularly in areas such as surgical procedures, physician assistance, health monitoring, and patient management. Numerous studies have confirmed the important practical value of such devices in improving medical personnel’s response efficiency, enhancing operational accuracy, and alleviating patient anxiety and pain^17,143–153^. For instance, in emergency scenarios, a study in Thailand showed that using smart glasses to assess the number of 11–30 casualties resulted in a nearly 9 percentage point improvement in accuracy (98.0% vs 89.2%) and reduced assessment time by over 50%^154^. In pediatric surgery, the use of VR glasses reduced the treatment time by an average of 5.53 min, significantly alleviating the anxiety and pain experienced by children throughout the treatment process^62^. Smart glasses equipped with AR and eye-tracking technology achieved high-precision eye tracking at a distance of 1 meter, with an accuracy of 1.0° and a margin of error controlled within ±0.1°, paving the way for new possibilities in health monitoring and medical diagnosis^49^. Additionally, in anesthesiology, AR smart glasses-assisted pediatric radial artery cannulation, increasing the success rate of interns from 71.7% to 89.8%, with a more than 23 percentage point reduction in complication rates^144^. For patients with Parkinson’s disease, the gait parameters obtained through AR glasses showed excellent reliability (ICC > 0.942)^108^. In neurosurgical procedures, the use of mixed reality navigation systems helped control the deviation in the location of lesions from standard navigation within 5.0 mm in 81.1% of cases^152^. Furthermore, smart glasses have shown potential in diet recognition. For example, the DietGlance system achieved an F1 score of 0.972 for dietary recognition and rigorously compared the estimated results of key constant nutrients with expert assessments. The analysis found that the average absolute percentage error (MAPE) for key constant nutrient estimation was approximately 17.92%^155^. These achievements fully demonstrate the vast potential of smart glasses in improving medical efficiency and service quality.

However, the application of the technology is not flawless. In the field of pediatric dentistry, a study on children with moderate to severe hearing impairments showed that VR glasses had no significant effect on lowering heart rate or alleviating anxiety during pulpectomy treatment (all assessment indicators had *p*-values > 0.05)^156^. Another study involving children aged 4–6 also confirmed that VR technology did not effectively reduce anxiety and pain during dental treatments. A clinical comparison in dental implant surgeries showed that although AR glasses-assisted dynamic computer-aided systems improved the three-dimensional positioning parameters of implants, the differences were not statistically significant^108^. These studies suggest that there are still technological bottlenecks and limitations in the effectiveness of smart glasses in medical applications, which require further optimization and breakthroughs.

In order to address the limitations of existing smart glasses models and leverage their advantages in the medical and healthcare field, this paper proposes the concept of next-generation smart glasses technology, aiming to overcome the issue of statistical insignificance, which can be dissected into two core components.

The first concept is the Non-Humanoid Wearable Robotic Assistant. This assistant is designed as a 24/7 active health companion equipped with advanced AI^157^. This enables the device to continuously adapt to the user’s lifestyle habits and health requirements^158–160^. Continuous learning capability^161^ allows for increasingly precise understanding of individual patterns over time, thereby delivering personalized health recommendations and behavioral guidance. For instance, by monitoring physiological signals such as heart rate and body temperature in real-time, combined with environmental factors (e.g., weather conditions, geographic location) and daily activity patterns, the smart glasses can optimize exercise routines or provide timely reminders for rest^162^. Furthermore, this type of intelligent assistant is equipped with situational awareness capabilities, utilizing built-in cameras and other sensors to capture visual data and recognize the user’s environment, thereby tailoring its service provision accordingly.

And the second is the Synchronous Decision-Making and Auxiliary Suggestions. Employing Multi-modal Large Language Models^163^, next-generation smart glasses not only enhance the quality of real-time decision-making^164^ but also deliver personalized suggestions^165^ derived from aggregated life and dietary data. These devices transcend the functionality of traditional mobile phone applications, deeply integrating into the user’s daily visual experience to offer more natural and intelligent life guidance^166^. The crux of this system lies in its interactive nature—it serves as an active learning assistant rather than merely a passive display device, aiding users in making informed decisions, enhancing quality of life, and providing immediate feedback on health prevention measures.

Smart glasses transform collected data into valuable insights, such as generating personalized nutritional plans based on the analysis of meal timing, food types, and physical activity levels, or suggesting optimal rest periods aligned with social activities to maintain peak performance. Moreover, these devices can seamlessly integrate with other health monitoring tools (e.g., smartwatches, glucose monitors) to form a comprehensive personal health ecosystem. To ensure the efficacy and safety of these recommendations, smart glasses regularly update their internal knowledge bases and reference the latest medical research and clinical guidelines. For elderly users, this technology is particularly beneficial as it aids in chronic disease management, enhances independent living capabilities^167^, and reduces the risk of accidental injuries, promoting longer and healthier aging. Additionally, smart glasses can connect with healthcare professionals, alerting doctors to potential health risks detected for prompt intervention^168^.

**Fig. 8:**
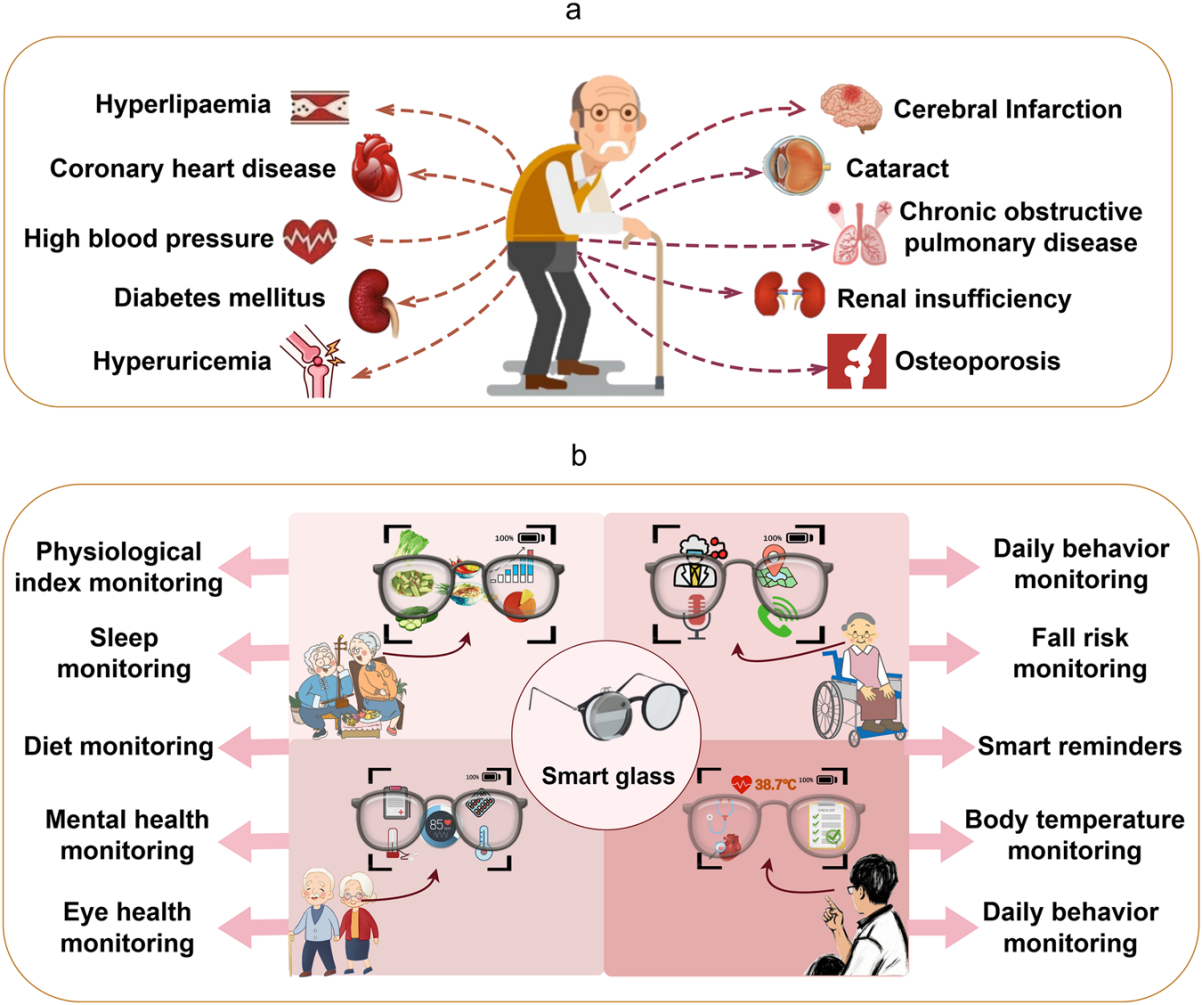
Next-Generation Smart Glasses. Enhancing elderly health management through non-humanoid wearable AI and synchronous decision support. **a** Health problems faced by the elderly. **b** Possible scenarios and ways for the elderly to use smart glasses (By Figdraw).

Figure 8 illustrates how smart glasses can serve as an integral part of elderly health management, offering continuous support and personalized health guidance that adapts to the unique needs of each user. The goal of this technology is to create an intelligent system characterized by the resonance of identity, vision, and senses across three screens, forming a non-humanoid multi-modal wearable robot^169^ that deeply integrates with the user’s life and health management. Smart glasses will serve as the control center for smart healthcare and other devices, allowing users to interact with these devices in real-time via voice recognition and gesture control, forming an efficient IoT ecosystem.

**Fig. 9:**
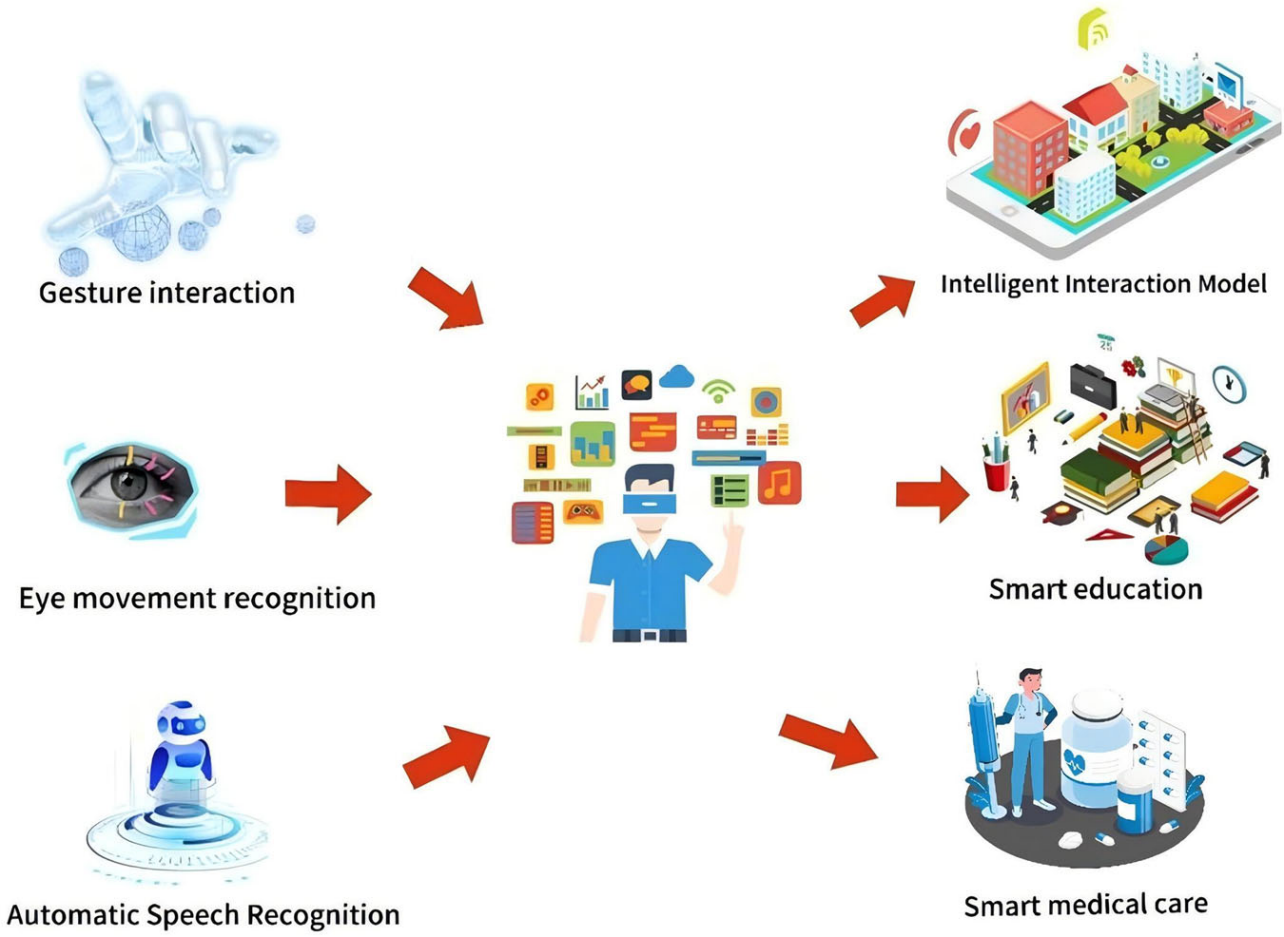
Application of multi-modal interaction paradigms across diverse scenarios. Technologies such as gesture interaction, eye-movement recognition, and automatic speech recognition can be applied to construct intelligent interaction models and play a role in fields such as intelligent education and intelligent healthcare.

The future of smart glasses will see significant advancements in multi-modal interaction technologies, which combine various forms of input and output—such as voice recognition, gesture control, haptic feedback, and AR—to create a more intuitive and efficient user interface. These technologies enable users to interact naturally with their devices without needing to rely on traditional input methods like keyboards or touchscreens. Figure 9 illustrates the application of different interaction modes across multiple scenarios, highlighting how these technologies can be seamlessly integrated into everyday life.

Voice recognition technology will continue to improve, allowing for more accurate and contextually aware interactions. Users can issue complex commands or queries, and the AI assistant within the smart glasses can respond appropriately, even in noisy environments. Gesture control will evolve to support a wider range of movements, enabling users to navigate menus, select options, or initiate actions through simple hand motions. Haptic feedback, the use of tactile sensations, will provide additional layers of interactivity, confirming user inputs or signaling alerts in a non-intrusive manner. AR overlays will enrich the user’s perception of the world, superimposing useful information directly onto the physical environment^170^, enhancing both productivity and entertainment. In terms of emotion recognition and situational understanding, the future multi-modal interaction system will be able to combine multiple input signals such as voice, facial expressions, and body movements to accurately identify the user’s emotional state^171^ and adjust the interaction strategy accordingly. For example, in the field of mental health management and healthcare, the system can identify mood swings based on the user’s facial expressions, tone of voice, and physiological signals to provide personalized intervention recommendations. Multi-modal Sentiment Analysis^172^ capabilities will greatly enhance the emotional connection between users and the system, making the interaction more humane and intelligent.

Ultimately, the goal of this technology is to create an intelligent system characterized by the seamless resonance of identity, vision, and senses across multiple interfaces, forming a non-humanoid multi-modal wearable robot that deeply integrates with the user’s life and health management. Smart glasses will serve as the central hub for smart healthcare and other connected devices, allowing users to interact with them in real-time via voice and gesture control, thereby establishing an efficient IoT ecosystem that supports a healthier, more connected lifestyle.

Despite the rapid advancements in smart glasses technology, several critical challenges remain to be addressed for enhancing functionality and user experience. These issues span across hardware optimization, energy management, interaction paradigms, materials science, interdisciplinary collaboration, and clinical validation.

Primarily, hardware optimization plays a crucial role. The processors integrated into smart glasses must facilitate real-time multitasking capabilities, encompassing AR/VR rendering, speech recognition, and execution of AI models. Nevertheless, the compact design of these devices imposes significant constraints on thermal dissipation and power consumption. Future research should therefore prioritize the development of low-power, high-performance chips specifically tailored for smart glasses. Collaborative efforts between software and hardware engineers are essential to enhance energy efficiency, ensuring that computational tasks are executed with optimal power utilization.

Secondly, effective energy management is imperative. The requirement for prolonged usage demands substantial battery longevity, a challenge not adequately addressed by existing lithium-ion batteries due to limitations in volume and capacity. Innovations in battery materials, such as solid-state or graphene-based technologies, offer potential improvements in energy density and safety. Modular battery designs that allow for easy replacement or integration with external power modules could provide users with enhanced flexibility. Moreover, solar charging presents an eco-friendly solution; however, advancements in photovoltaic materials, particularly perovskite cells, are necessary to increase power generation efficiency within the confined surface area of smart glasses frames. Hybrid power systems that combine multiple power sources could ensure an efficient and uninterrupted power supply, addressing the unique challenges posed by wearable technology.

As interaction paradigms, traditional touch and key controls are gradually being replaced by more intuitive interactions, such as gestures, eye movements, and brain signals. Flexible sensors and emerging interface technologies cater to user needs for convenience and efficiency while promoting a shift from “command” to “perception”-based interaction models. Yet, existing gestures and voice controls may falter in complex environments. Future development should introduce advanced gesture tracking algorithms and noise-resistant speech recognition technologies. Integrating multi-modal interactions—combining gestures, voice, touch, and eye tracking—will offer a more seamless user experience, reducing latency and ensuring smooth operation.

For materials, the exploration of flexible materials presents a natural way to interact and significantly improves wearing comfort and functional integration. Flexible smart wrist guards can enable gesture recognition through capturing subtle hand movements, whereas eye-tracking technology facilitates touchless interaction. In complex scenarios, multi-modal combinations enhance interaction efficiency. Furthermore, brain-computer interfaces (BCIs)^173^ that interpret EEG signals into operational commands represent a breakthrough in traditional interaction methods, offering inclusive solutions for individuals with physical disabilities^174^.

For clinical validation and efficacy evaluation, critical to assessing the effectiveness of smart glasses technology is clinical validation and efficacy evaluation. Future studies should prioritize large-scale clinical trials and real-world data analysis to verify the impact of smart glasses on user health management. By establishing a robust evidence base, these evaluations will facilitate widespread acceptance and adoption of smart glasses in healthcare settings and beyond.

**Fig. 10:**
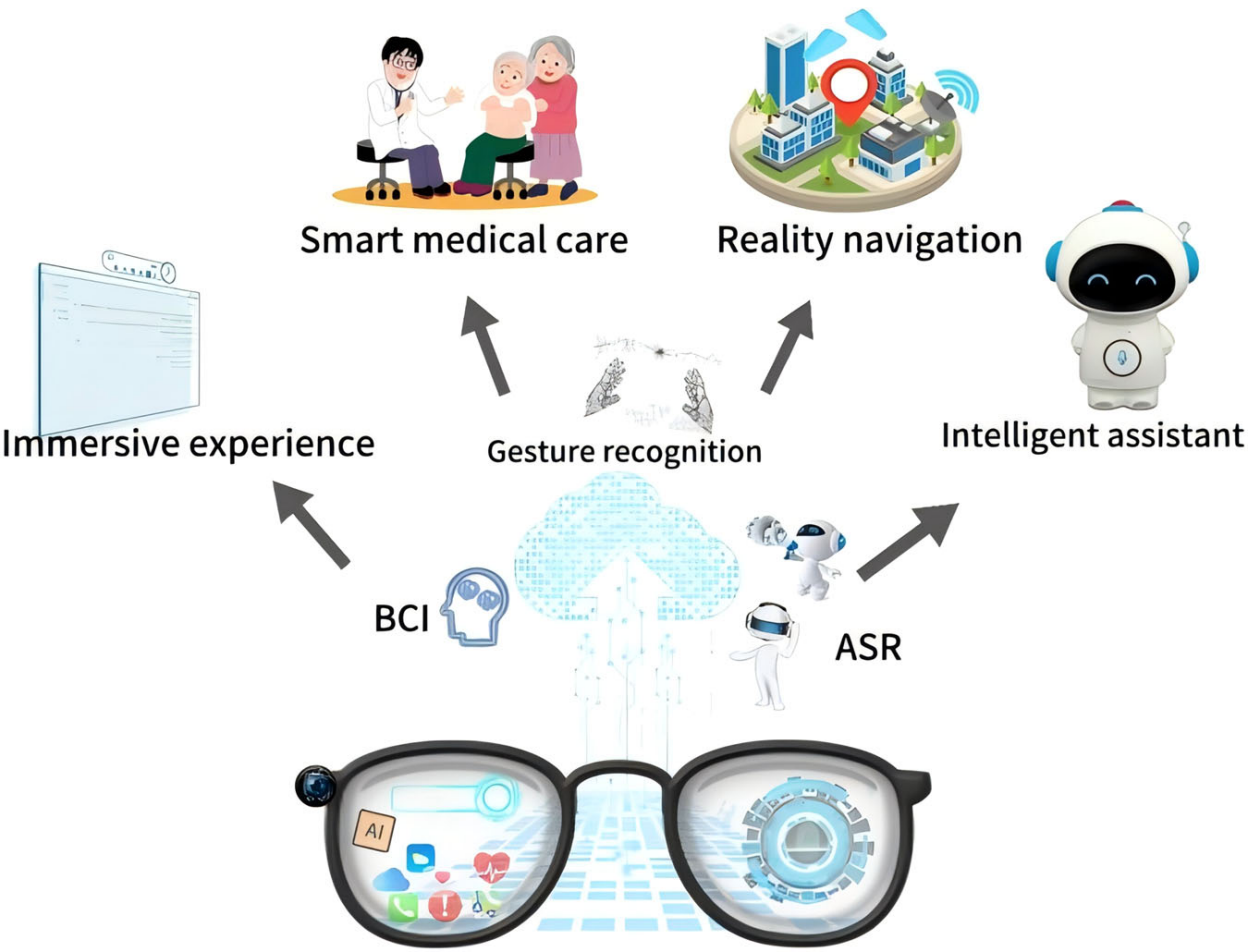
Prediction for the future development of smart glasses. Smart glasses leverage technologies such as BCI, ASR, and gesture recognition to achieve functions such as immersive experience, smart medical care, reality navigation, and intelligent assistant.

The future development of smart glasses is expected to align with the trends illustrated in Fig. 10, enabling their widespread application across various scenarios and establishing them as intelligent health assistants for personalized and real-time health management and interaction.

This study reveals the significant potential of smart glasses in digital health management. By integrating IoT and AI technologies, smart glasses have not only achieved real-time health monitoring, chronic disease management, and personalized interventions but also markedly enhanced the speed and accuracy of medical responses. The research finds that the application of smart glasses has transcended traditional monitoring boundaries, will become an effective tool to promote treatment adherence and improve quality of life. Despite challenges such as data synchronization efficiency, hardware comfort, and adaptability, smart glasses are emerging as integral components of personal health ecosystems. They are progressively transforming paradigms of health management and disease prevention, paving the way for more intelligent and personalized healthcare services. Future research should focus on conducting large-scale clinical trials to validate these findings across diverse populations. Additionally, exploring the long-term impact of continuous health monitoring via smart glasses will be crucial for understanding their full potential in transforming healthcare delivery.

## Methods

To systematically identify relevant literature on the application of smart glasses in healthcare and health management, we developed a comprehensive and rigorous search strategy to ensure high recall, relevance, and reproducibility.

### Search type and conceptual framework

We adopted a Boolean search approach that combined both controlled vocabulary, such as MeSH terms, and free-text keywords to accommodate indexing variations across multiple databases. The search strategy was constructed around two core conceptual domains: the technology of interest, which included terms like “smart glasses,” “AR glasses,” “VR glasses,” “AI glasses,” and “intelligent glasses,” and the application domain, encompassing terms such as “health management,” “healthcare,” “medical,” and “clinical.” The final search string applied consistently across all databases was: (“smart glasses” OR “AR glasses” OR “VR glasses” OR “AI glasses” OR “intelligent glasses”) AND (“health management” OR “healthcare” OR “medical” OR “clinical”).

### Databases and search scope

To focus on the latest advancements in artificial intelligence and smart glasses, we conducted a literature search in three major academic databases—PubMed, Web of Science Core Collection, and IEEE Xplore—retrieving English-language publications from January 2021 to April 2025 to ensure comprehensive coverage of both medical and engineering fields. PubMed was used to identify biomedical and health-related studies, yielding 534 records; the Web of Science Core Collection provided access to multidisciplinary research, including clinical and engineering studies, with 276 records identified; IEEE Xplore focused on literature related to smart hardware and human-computer interaction, contributing 51 records.

### Screening and reference management

All identified records were imported into EndNote reference management software for deduplication.

### Inclusion and exclusion criteria

The inclusion criteria for this review required studies that explicitly investigated smart glasses or their variants, such as AR, VR, or AI glasses, while excluding those that focused on generalized wearable devices. Eligible studies were those centered on healthcare or medical-related applications, including but not limited to health monitoring, clinical decision support, telemedicine, rehabilitation, nursing care, or disease screening. Studies were excluded if they were duplicate publications across databases (in such cases, only the most complete version was retained), focused on non-glasses wearable devices such as smartwatches or wristbands, or addressed non-healthcare-related contexts such as industrial, military, or entertainment applications. Additionally, non-peer-reviewed literature, including preprints, abstracts without full-text availability, and unpublished theses, was excluded to ensure the scientific rigor and credibility of the included studies.

## Supplementary information

PRISMA_2020_checklist (1)

## Data Availability

No datasets were generated or analyzed during the current study.

## Abbreviations

5G: 5G Networks
AI: Artificial Intelligence
AR: Augmented Reality
CDSS: Clinical Decision Support System
DL: Deep Learning
EC: Edge Computing
EHR: Electronic Health Records
FSPB: Flexible Self-Powered Bioelectronics
IoMT: Internet of Medical Things
LLM: Large Language Models
MR: Mixed Reality
NLP: Natural Language Processing
OS: Operating System
PHM: Proactive Health Management
PHR: Personal Health Records
RPM: Remote Patient Monitoring
RTHM: Real-Time Health Monitoring
SVM: Support Vector Machines
UI: User Interface
UX: User Experience
VR: Virtual Reality
